# Noninvasive prediction of node-positive breast cancer response to presurgical neoadjuvant chemotherapy therapy based on machine learning of axillary lymph node ultrasound

**DOI:** 10.1186/s12967-023-04201-8

**Published:** 2023-05-21

**Authors:** Hao Zhang, Wen Cao, Lianjuan Liu, Zifan Meng, Ningning Sun, Yuanyuan Meng, Jie Fei

**Affiliations:** 1 Department of Spinal Surgery, The Affiliated Hospital of Qingdao University, Qingdao, Shandong China; 2 Department of Medical Record Management, The Affiliated Hospital of Qingdao University, Pingdu District, Qingdao, Shandong China; 3 Department of Ultrasound, Qingdao Hospital, University of Health and Rehabilitation Sciences (Qingdao Municipal Hospital), Qingdao, Shandong China; 4 Department of Blood Transfusion, The Affiliated Hospital of Qingdao University, Qingdao, Shandong China; 5 Department of Breast Disease Center, The Affiliated Hospital of Qingdao University, Qingdao, Shandong China; 6 Department of Cardiac Ultrasound, The Affiliated Hospital of Qingdao University, Qingdao, Shandong China; 7 Department of Breast Imaging, The Affiliated Hospital of Qingdao University, No. 59 Haier Road, Qingdao, 266000 Shandong China

**Keywords:** Radiomics, Ultrasonography, Lymph nodes, Neoadjuvant therapy

## Abstract

**Objectives:**

To explore an optimal model to predict the response of patients with axillary lymph node (ALN) positive breast cancer to neoadjuvant chemotherapy (NAC) with machine learning using clinical and ultrasound-based radiomic features.

**Methods:**

In this study, 1014 patients with ALN-positive breast cancer confirmed by histological examination and received preoperative NAC in the Affiliated Hospital of Qingdao University (QUH) and Qingdao Municipal Hospital (QMH) were included. Finally, 444 participants from QUH were divided into the training cohort (n = 310) and validation cohort (n = 134) based on the date of ultrasound examination. 81 participants from QMH were used to evaluate the external generalizability of our prediction models. A total of 1032 radiomic features of each ALN ultrasound image were extracted and used to establish the prediction models. The clinical model, radiomics model, and radiomics nomogram with clinical factors (RNWCF) were built. The performance of the models was assessed with respect to discrimination and clinical usefulness.

**Results:**

Although the radiomics model did not show better predictive efficacy than the clinical model, the RNWCF showed favorable predictive efficacy in the training cohort (AUC, 0.855; 95% CI 0.817–0.893), the validation cohort (AUC, 0.882; 95% CI 0.834–0.928), and the external test cohort (AUC, 0.858; 95% CI 0.782–0.921) compared with the clinical factor model and radiomics model.

**Conclusions:**

The RNWCF, a noninvasive, preoperative prediction tool that incorporates a combination of clinical and radiomics features, showed favorable predictive efficacy for the response of node-positive breast cancer to NAC. Therefore, the RNWCF could serve as a potential noninvasive approach to assist personalized treatment strategies, guide ALN management, avoiding unnecessary ALND.

**Supplementary Information:**

The online version contains supplementary material available at 10.1186/s12967-023-04201-8.

## Introduction

Breast cancer is a major public health issue, and more than 4.4 million women’s health is threatened by breast cancer worldwide [1]. Since 1894, axillary lymph node (ALN) dissection (ALND) has been regarded as an integral part of surgical treatment and applied in all breast cancer surgeries [2]. However, there are many potential complications of ALND, including postoperative arm pain, nerve injury, lymphedema and significant trauma, limiting its further application [3]. Recently, neoadjuvant chemotherapy (NAC) has been regarded as the preoperative initial systemic treatment and applied for patients with clinically node-positive breast cancer subtypes to improve survival [4, 5]. According to previous studies, more than 50% of patients could achieve pathological complete response (pCR) post-NAC, avoid ALND and receive conservation surgery [6, 7]. However, in the clinic, invasive operations such as ALND and sentinel ALN biopsy, are still regarded as routine methods for assessing the status of the ALN [8].

Therefore, many studies have tried to assess ALN status based on noninvasive approaches, such as clinical prediction models, magnetic resonance imaging (MRI) and ALN ultrasound (ALNU), to reduce unnecessary ALND [9–13]. However, these preoperative prediction models have not yet been used in large-scale clinical practice because of the limited power of these traditional clinical and single imaging characteristics. With the development of radiomics, many radiomics models have shown favorable predictive efficacy and have been applied in clinical decision-making [14, 15]. For example, Dong et al. [16] used the least absolute shrinkage and selection operator (LASSO) and stepwise multivariate logit regression to achieve ultrasound radiomics feature selection and build the model to predict the histological grades and Ki-67 expression of hepatocellular carcinoma. To the best of our knowledge, there have been only limited reports of predictive models combining clinical characteristics and radiomics features to assess ALN status after NAC.

Therefore, we aimed to explore an optimal model to predict ALN status and assess the response of patients with node-positive breast cancer to presurgical NAC with machine learning (ML) using clinical and ALNU-based radiomic features.

## Methods

### Population

In this study, 1014 patients with ALN-positive breast cancer confirmed by histological examination and received preoperative NAC in the Affiliated Hospital of Qingdao University (QUH) and Qingdao Municipal Hospital (QMH) from July 2016 to September 2022 were initially included. Institutional review board approval of the two hospitals (QUH and QMH) was obtained, and patient informed consent was waived for this retrospective analysis.

**Fig. 1:**
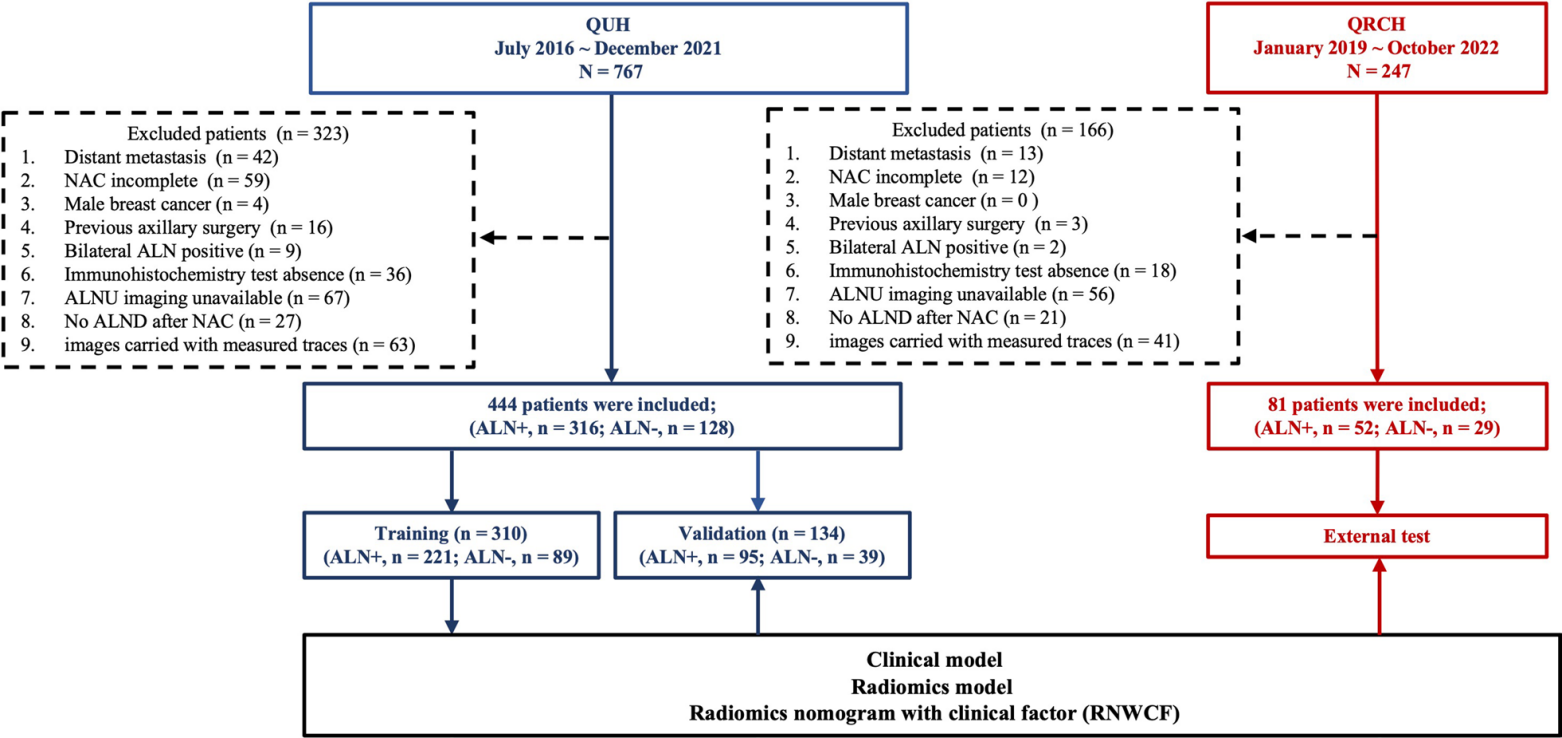
The detailed flowchart for patient inclusion

The exclusion criteria were patients with distant metastasis (n = 55); NAC incomplete due to lesion augmentation (n = 71); male breast cancer (n = 4); previous axillary surgery (n = 19); bilateral ALN positivity (n = 11); immunohistochemistry test absence (n = 54); ALNU imaging unavailability (or more than 4 weeks before surgery) (n = 123); no ALND after NAC (n = 48); and images carried with measured traces (which would cause interference to radiomic features extraction (n = 104). The flowchart of inclusion and exclusion is illustrated in Fig. 1.

Finally, a total of 525 patients were enrolled, and 444 patients from QUH were divided into the training cohort (n = 310) and validation cohort (n = 134) based on the date of ALNU examination to train and validate the ML models. 81 patients from QMH were divided into the external test cohort and used to evaluate the external generalizability of our prediction models.

### Diagnosis of ALN status

The final diagnosis of ALN status in all patients was established based on the pathological results of ALND, and the pathological results were confirmed by at least 2 pathologists with more than 5 years of experience.

### Clinical and pathologic characteristics

Clinical data and pathologic characteristics were drawn from medical records. The NAC regimens included taxane plus anthracycline and cyclophosphamide (TAC), anthracycline plus cyclophosphamide followed by taxane (AC-T), anthracycline plus taxane (AT), anthracycline plus cyclophosphamide followed by taxane plus trastuzumab (AC-TH), anthracycline plus cyclophosphamide followed by taxane plus trastuzumab and pertuzumab (AC-THP), taxane plus trastuzumab (TH), taxane plus trastuzumab and pertuzumab (THP), taxane plus carboplatin (TCb), taxane plus carboplatin and trastuzumab (TCbH), and taxane and carboplatin plus trastuzumab and pertuzumab (TCbHP). In addition, the details of NAC regimens and course are shown in the Additional file 1.

The status of estrogen receptor (ER), human epidermal growth factor receptor 2 (HER2), and Ki-67 was assessed based on the immunohistochemical staining of breast tumors. The definition of ER-positive (≥ 10% immunostained cells) and HER2 positive (≥ 3+ in hematoxylin–eosin staining, or 2+ with confirmation of HER2 gene amplification by fluorescence in situ hybridization) has been widely reported in previous studies [17]. In the present study, Ki-67 with a proliferation index higher than 20% was considered positive.

### ALNU examination

All patients from QUH were using a 5–12 MHz linear-array transducer (Hitachi-EUB7500) or a 5–14 MHz linear-array transducer (Siemens S2000), and ALNU was performed at the end of NAC for response evaluation by four radiologists with 3–15 years of experience.

Patients from QMH were using a 6- to 15-MHz linear-array transducer (GE Logic quasi E9), 5–12 MHz linear-array transducer (Philips EPIQ7) or 5−4 MHz (Hitachi ALOKA), and ALNU was performed at the end of NAC for response evaluation by three radiologists with 4, 7, and 12 years of experience, respectively.

### Evaluation of ALNU

**Fig. 2:**
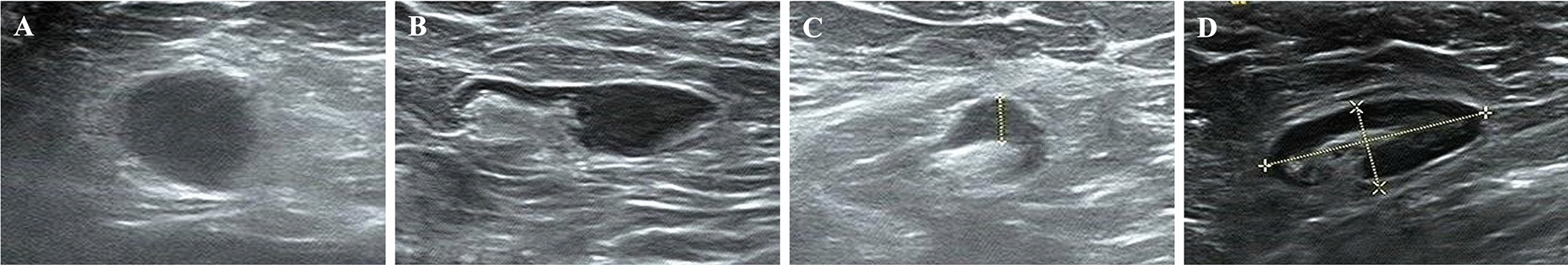
Radiological characteristics of the ALN. **A** The absence of echogenic fatty hilum; **B** focal cortical thickening; **C** the measurement of node cortical thickness; **D** the measurement for long axis and short axis of axillary nodes

The most suspicious ALN was selected and reviewed by two radiologists with 7–10 years of experience in sonography diagnosis who were unknown to surgical and pathological reports with node size and shape measured and evaluated. Hypoechoic cortex and hyperechoic medulla next to it are major features to recognize ALN. The thicker the cortex was, the greater probability of residue disease considered to be. If an ALN presented focal cortical thickening or absence of the echogenic fatty hilum, then it was recorded [18, 19]. When a disagreement occurred, another radiologist participated in the evaluation until a consensus was reached. The ALN maximum long axis was regarded as the long axis, and the perpendicular axis was regarded as the short axis. The ALN axis and cortical thickness were measured well due to the software in the working station with the help of a reference provided by the scale plate from primary images (Fig. 2).

### Construction of the clinical model

The significant factors selected in univariable analysis were further incorporated into the multiple analysis. Then, the backward stepwise regression analysis was performed to determine the independent risk factors and build the clinical factor model. Odds ratios (OR) as estimates of relative risk with 95% confidence intervals (CI) were calculated for each independent risk factor.

### ALN segmentation

**Fig. 3:**
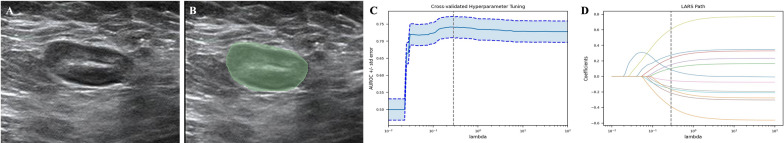
Construction of radiomics signatures. **A**, **B** ROI segmentation; **C** Cross-validation parameter tuning parameter. The optimal values of the LASSO tuning parameter are indicated by the dotted vertical lines; **D** LASSO coefficient profiles of the radiomics features. A coefficient profile plot was generated versus the selected value using tenfold cross-validation; the vertical line was plotted with selected radiomics features

The ROI was segmented manually by two radiologists with at least 10 years of experience from QUH and QMH who were blinded to the ALN status using 3DSlicer software (version 4.8.1). The ROI was drawn along the outline of most suspicious ALN to achieve accurate segmentation and include the whole lesion (Fig. 3A, B).

### Radiomics feature extraction

All radiomics features were extracted from the ROI using the PyRadiomics package (based on Python). 1032 radiomic features of each ROI were extracted using gray level co-occurrence matrix (GLCM), gray level dependence matrix (GLDM), gray level run length matrix (GLRLM), and gray level size zone matrix (GLSZM). In addition, the details of the extraction method and radiomic features are shown in the Additional file 1.

### Construction of the radiomics model

Z-score normalization of radiomics features was performed based on the mean and standard deviation from the data. For our high-dimensionality dataset, feature selection and classification were necessary, so the minimal redundancy maximum relevance (MRMR) algorithm was applied to evaluate the feature relevance. The MRMR selected subset of features was enrolled in LASSO regression model to select the most valuable features and build the radiomics model in the training cohort. Fivefold cross-validation was set to finalize candidate features. In addition, the radiomics score for each patient was calculated by the selected features based on their weighted coefficients.

### Development of the radiomics nomogram with clinical factors (RNWCF)

The RNWCF was developed by combining the significant clinical factors and radiomics score. The independent risk factors were determined, and the RNWCF was built based on logistic regression analysis.

### Evaluation of the performance of different models

For the validation cohort and external test cohort, the accuracy, sensitivity, and specificity of different models were calculated. Receiver operating characteristic (ROC) curves and the area under the ROC curve (AUC) were used to assess the performance of the models and describe their predictive power.

### Statistical analysis

Normally distributed continuous variables are shown as the mean ± standard deviation, and categorical variables are shown as percentages (%). Student’s t test, the Chi-square test and Fisher’s exact test were performed by SPSS Statistics software, version 22.0 (SPSS Inc., Chicago, IL, USA). Building and evaluating the performance of different models (including ROC curve analysis, logistic regression analysis, MRMR analysis, LASSO regression, etc.) were performed by Python 3.10.4. The factors with P values < 0.05 in univariable analysis were selected and incorporated into the multiple analysis. P < 0.1 (two-sided) was considered statistically significant in the multiple analysis.

## Results

### Population characteristics

As shown in Table 1 and 310 patients were enrolled in the training cohort according to the screening criteria, including 221 ALN+ (mean age, 49.74 ± 10.88 years; 63.35% ER+) and 89 ALN− patients (mean age, 49.21 ± 9.76 years; 50.56% ER+); and 134 patients were enrolled in the validation cohort, including 95 ALN+ (mean age, 50.42 ± 9.82 years; 69.47% ER+) and 39 ALN− patients (mean age, 49.54 ± 9.31 years; 46.15% ER+). 81 patients (52 ALN+ and 29 ALN−) from QMH were enrolled in the external test cohort. In addition, there was no significant difference in NAC regimens between ALN+ and ALN− patients in the three cohorts (P > 0.05, respectively).

**Table 1:** Demographic and clinical characteristics of patients

Characteristic	Training cohort (N = 310)			Validation cohort (N = 134)			External test cohort (N = 81)		
	ALN+ (N = 221)	ALN− (N = 89)	P	ALN+ (N = 95)	ALN− (N = 39)	P	ALN+ (N = 52)	ALN− (N = 29)	P
Age (years)	49.74 ± 10.88	49.21 ± 9.76	0.691	50.42 ± 9.82	49.54 ± 9.31	0.632	50.63 ± 9.31	50.00 ± 9.55	0.771
Pathological type, No (%)			0.644			0.657			1.000
IDC^a^	207 (93.67)	81 (91.01)		85 (89.47)	37 (94.87)		51 (98.08)	29 (100.00)	
ILC^b^	5 (2.26)	3 (3.37)		3 (3.16)	0 (0.00)		1 (1.92)	0 (0.00)	
Others	9 (4.07)	5 (5.62)		7 (7.37)	2 (5.13)		0 (0.00)	0 (0.00)	
Node long axis (cm)	1.45 ± 0.68	1.24 ± 0.54	0.008	1.38 ± 0.65	1.21 ± 0.52	0.147	1.59 ± 0.75	1.47 ± 0.58	0.436
Node short axis (cm)	0.74 ± 0.38	0.55 ± 0.18	< 0.001	0.72 ± 0.40	0.53 ± 0.18	0.003	0.82 ± 0.35	0.70 ± 0.28	0.121
Cortical thickness (cm)	0.50 ± 0.60	0.19 ± 0.12	< 0.001	0.52 ± 0.51	0.20 ± 0.12	< 0.001	0.49 ± 0.34	0.22 ± 0.22	< 0.001
Cortical thickening, No (%)			< 0.001			0.310			0.549
Yes	39 (17.65)	3 (3.37)		17 (17.89)	4 (10.26)		3 (5.77)	0 (0.00)	
No	182 (82.35)	86 (96.63)		78 (82.11)	35 (89.74)		49 (94.23)	29 (100.00)	
Medulla absence, No (%)			< 0.001			0.054			0.001
Yes	45 (20.36)	3 (3.37)		27 (28.42)	5 (12.82)		19 (36.54)	1 (3.45)	
No	176 (79.64)	86 (96.63)		68 (71.58)	34 (87.18)		33 (63.46)	28 (96.55)	
T stage, No (%)			0.551			0.024			0.009
T1	17 (7.69)	5 (5.62)		7 (7.37)	5 (12.82)		14 (26.92)	9 (31.03)	
T2	130 (58.82)	47 (52.81)		62 (65.26)	17 (43.59)		26 (50.00)	19 (65.52)	
T3	64 (28.96)	31 (34.83)		22 (23.16)	10 (25.64)		12 (23.08)	0 (0.00)	
T4	10 (4.52)	6 (6.74)		4 (4.21)	7 (17.95)		0 (0.00)	1 (3.45)	
N stage, No (%)			< 0.001			0.001			< 0.001
N1	140 (63.35)	78 (87.64)		60 (63.16)	37 (94.87)		25 (48.08)	26 (89.66)	
N2	43 (19.46)	10 (11.24)		18 (18.95)	1 (2.56)		11 (21.15)	3 (10.34)	
N3	38 (17.19)	1 (1.12)		17 (17.89)	1 (2.56)		16 (30.77)	0 (0.00)	
ER status, No (%)			0.038			0.011			0.401
Positive	140 (63.35)	45 (50.56)		66 (69.47)	18 (46.15)		37 (71.15)	18 (62.07)	
Negative	81 (36.65)	44 (49.44)		29 (30.53)	21 (53.85)		15 (28.85)	11 (37.93)	
HER2 status, No (%)			0.006			0.034			0.031
Positive	84 (38.01)	43 (48.31)		28 (29.47)	19 (48.72)		16 (30.77)	16 (55.17)	
Negative	137 (61.99)	46 (51.69)		67 (70.53)	20 (51.28)		36 (69.23)	13 (44.83)	
Ki-67 > 20%, No (%)			0.010			0.016			0.576
Yes	116 (52.49)	61 (68.54)		44 (46.32)	27 (69.23)		32 (61.54)	16 (55.17)	
No	105 (47.51)	28 (31.46)		51 (53.68)	12 (30.77)		20 (38.46)	13 (44.83)	
NAC Regimens, No (%)			0.152			0.280			0.161
AC-T	41 (18.55)	21 (23.60)		16 (16.84)	5 (12.82)		9 (17.31)	4 (13.79)	
AC-TH	27 (12.22)	13 (14.61)		10 (10.53)	3 (7.79)		5 (9.62)	2 (6.90)	
AC-THP	11 (4.98)	2 (2.25)		1 (1.05)	1 (2.56)		0 (0.00)	0 (0.00)	
AT	22 (9.95)	3 (3.37)		18 (18.95)	6 (15.38)		6 (11.54)	1 (3.45)	
TAC	77 (34.84)	21 (23.60)		31 (32.63)	7 (17.95)		22 (42.31)	8 (27.59)	
TCb	5 (2.26)	3 (3.37)		3 (3.16)	2 (5.13)		0 (0.00)	0 (0.00)	
TCbH	7 (3.17)	5 (5.62)		4 (4.21)	3 (7.69)		1 (1.92)	0 (0.00)	
TCbHP	15 (6.79)	9 (10.11)		4 (4.21)	3 (7.69)		2 (3.85)	2 (6.90)	
TH	2 (0.90)	1 (1.12)		1 (1.05)	0 (0.00)		0 (0.00)	1 (3.45)	
THP	14 (6.33)	11 (12.36)		7 (7.37)	9 (23.08)		7 (13.46)	11 (37.93)	

^a^Invasive ductal carcinoma

^b^Invasive lobular carcinoma

### Clinical model

**Fig. 5:**
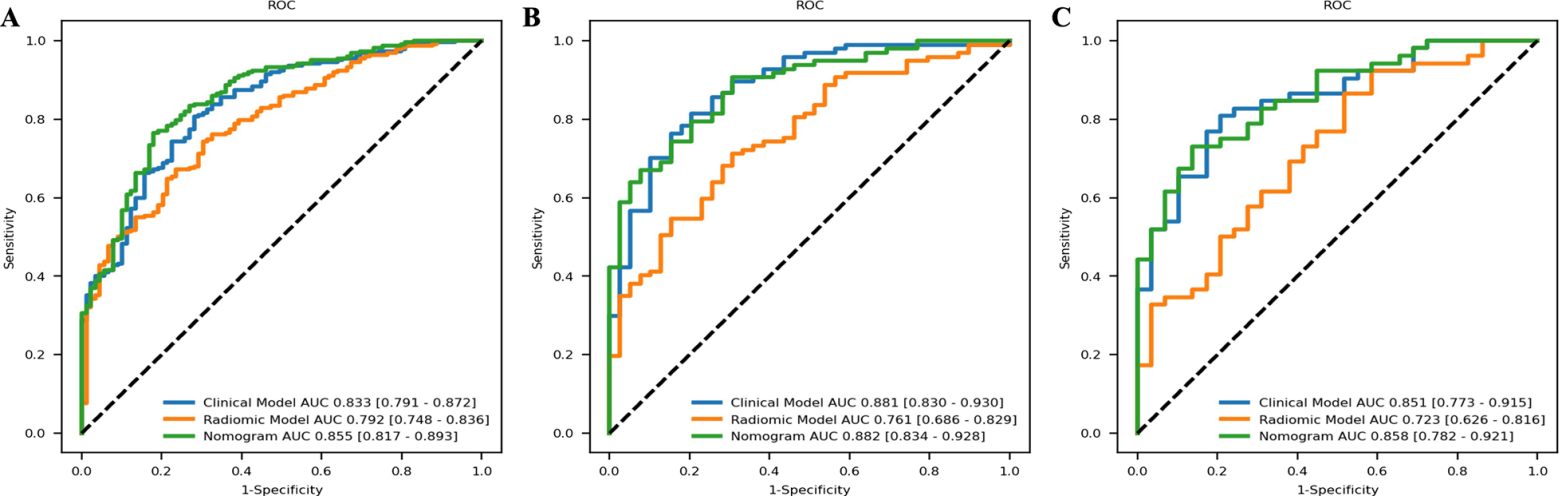
The ROC curves of the clinical model, radiomics model and RNWCF in **A** the training cohort, **B** the validation cohort and **C** the external test cohort. The ROC curves of the RNWCF nomogram are outperformed than the radiomics signature in both the validation and external test cohort

The results of univariate analysis of clinicopathological variables are shown in Table 2. Table 3 shows that the clinical nodal category (N stage) (P = 0.009, OR = 2.320), long axis (P = 0.056, OR = 0.453), short axis (P = 0.051, OR = 9.142), cortical thickness of ALN (P < 0.001, OR = 1308.975), ER (P = 0.046, OR = 1.883), and Ki-67 (P = 0.024, OR = 0.489) were proven to be independent clinical predictors in the training cohort and input into the model. The AUCs of the clinical model in the training cohort (0.833; 95% CI 0.791–0.872), validation cohort (0.881; 95% CI 0.830–0.930) and external test cohort (0.851; 95% CI 0.773–0.915) are shown in Fig. 5.

**Table 2:** Univariate analysis of clinical variables

Variable	OR	[0.025	0.975]	S.E.	Z	P
Age	1.005	0.982	1.028	0.012	0.425	0.671
T stage	0.767	0.536	1.096	0.182	− 1.453	0.146
N stage	3.246	1.868	5.641	0.282	4.179	0.000
Long axis (mm)	1.801	1.158	2.798	0.225	2.616	0.009
Short axis (mm)	16.263	4.559	58.032	0.649	4.298	0.000
Cortical thickness (mm)	2926.025	191.330	44712.125	1.391	5.736	0.000
Cortical thickening	6.109	1.837	20.328	0.613	2.951	0.003
Medulla absence	7.492	2.266	24.779	0.610	3.300	0.001
ER	1.702	1.036	2.798	0.254	2.096	0.036
HER2	0.497	0.302	0.818	0.254	− 2.753	0.006
Ki-67	0.502	0.299	0.845	0.265	− 2.599	0.009
NAC Regimens	0.957	0.875	1.048	0.046	− 0.945	0.345

**Table 3:** Backward-stepwise regression of clinical variables (clinical model)

Variable	OR	[0.025	0.975]	S.E.	Z	P
N stage	2.320	1.235	4.358	0.322	2.614	0.009
Long axis (mm)	0.453	0.201	1.020	0.414	− 1.911	0.056
Short axis (mm)	9.142	0.990	84.437	1.134	1.951	0.051
Cortical thickness	1308.975	82.023	20889.460	1.413	5.078	0.000
ER	1.883	1.013	3.501	0.317	2.000	0.046
Ki67	0.489	0.263	0.910	0.317	− 2.255	0.024

### Radiomics feature selection and model building

As shown in Table 4; Fig. 3, the nine most valuable radiomics features were selected and used to build the radiomics model. The radiomics signatures were calculated based on the coefficients of the radiomics features (Table 5).

**Table 4:** The selected radiomic features

Variable	OR	[0.025	0.975]	S.E.	Z	P
lbp-2D_gldm_DependenceEntropy	0.573	0.382	0.860	1.230	0.068	0.003
lbp-2D_glszm_GrayLevelNonUniformityNormalized	1.411	0.987	2.020	1.201	6.593	0.026
original_shape2D_Elongation	1.258	0.926	1.709	1.170	4.332	0.062
log-sigma-0-2-mm-3D_glcm_Correlation	0.728	0.542	0.978	1.163	0.122	0.015
wavelet-LL_glszm_LargeAreaHighGrayLevelEmphasis	0.826	0.642	1.062	1.137	0.225	0.059
wavelet-LL_glcm_MCC	2.142	1.530	3.001	1.188	84.099	0.000
lbp-2D_glszm_SizeZoneNonUniformityNormalized	0.792	0.580	1.080	1.171	0.229	0.061
wavelet-LL_firstorder_Skewness	1.421	0.999	2.020	1.197	7.071	0.022
wavelet-LH_glcm_MCC	0.746	0.546	1.019	1.172	0.159	0.029

**Table 5:** Formula for calculation of radiomic signatures

Variable	Coefficients
lbp-2D_gldm_DependenceEntropy	− 0.556
lbp-2D_glszm_GrayLevelNonUniformityNormalized	0.345
original_shape2D_Elongation	0.230
log-sigma-0-2-mm-3D_glcm_Correlation	− 0.317
wavelet-LL_glszm_LargeAreaHighGrayLevelEmphasis	− 0.191
wavelet-LL_glcm_MCC	0.762
lbp-2D_glszm_SizeZoneNonUniformityNormalized	− 0.233
wavelet-LL_firstorder_Skewness	0.351
wavelet-LH_glcm_MCC	− 0.293

The AUCs of the radiomics model were 0.792 (95% CI 0.748–0.836) in the training cohort, 0.761 (95% CI 0.686–0.829) in the validation cohort and 0.723 (95% CI 0.626–0.816) in the external test cohort (Fig. 5).

### Development of RNWCF

**Fig. 4:**
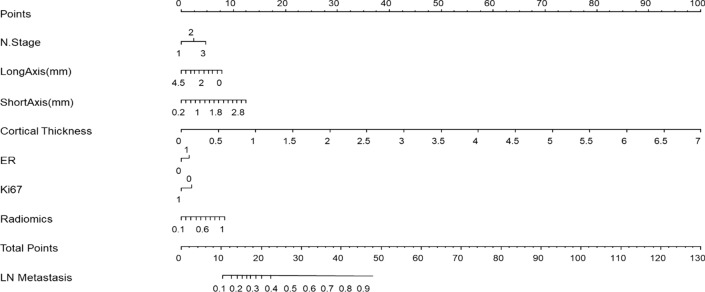
The RNWCF nomogram, combining N stage, long axis, short axis, cortical thickness, ER, Ki67 and radiomics signatures, developed in the training set

The N stage, long axis, short axis, cortical thickness of the ALN, ER, Ki-67, and radiomic signatures were incorporated into the construction of the RNWCF (Table 6; Fig. 4). Compared with the clinical model and radiomics model, the RNWCF showed the favorable AUCs in the training cohort (0.855; 95% CI 0.817–0.893), validation cohort (0.882; 95% CI 0.834–0.928) and external test cohort (0.858; 95% CI 0.782–0.921) (Fig. 5). In addition, the accuracy, sensitivity, and specificity of the different models are shown in Table 7.

**Table 6:** Multivariable logistic regression analysis of clinical variables and radiomic signatures (RNWCF)

Variable	OR	[0.025	0.975]	S.E.	Z	P
N stage	2.336	1.256	4.345	1.372	14.585	0.003
Long axis (mm)	0.533	0.228	1.245	1.542	0.233	0.063
Short axis (mm)	5.070	0.508	50.602	3.235	3.987	0.073
Cortical thickness	181.781	10.475	3152.654	4.289	35.659	0.000
ER	1.722	0.897	3.310	1.395	5.114	0.045
Ki67	0.483	0.251	0.928	1.395	0.113	0.013
Radiomics	28.974	5.474	153.393	2.340	52.405	0.000

**Table 7:** Diagnostic performances of the clinical model, radiomic model and RNWCF in the validation and external test cohorts

Data set	Models	Accuracy (95% CI)	Sensitivity (95% CI)	Specificity (95% CI)
Validation cohort	Clinical	0.809 (0.757–0.860)	0.814 (0.750–0.875)	0.795 (0.684–0.896)
	Radiomics	0.706 (0.640–0.772)	0.711 (0.630–0.781)	0.692 (0.564–0.821)
	RNWCF	0.846 (0.794–0.897)	0.907 (0.853–0.955)	0.692 (0.568–0.812)
External test cohort	Clinical	0.802 (0.716–0.877)	0.808 (0.708–0.893)	0.793 (0.656–0.920)
	Radiomics	0.728 (0.642–0.815)	0.865 (0.787–0.941)	0.483 (0.333–0.647)
	RNWCF	0.778 (0.691–0.852)	0.731 (0.623–0.830)	0.862 (0.750–0.963)

## Discussion

For breast cancer patients with initial positive nodes, the ALN status after NAC is used as an important reference for axilla treatment decision making and may possibly exempt ALND when a negative result is obtained. The diagnostic accuracy of current imaging modalities for ALN status assessment post-NAC is generally inferior to that pre-NAC [20]. Morphologic changes, such as atrophy and fibrosis, in ALN caused by the response to NAC are the main reasons that make it difficult to identify residual metastases [21]. As a result, many researchers focused on using clinical models to predict ALN status after NAC.

However, the diagnostic performance of radiomics model to assess ALN status after NAC has seldomly been reported in previous studies. In this study, three models (clinical model, radiomics model and RNWCF) were built to evaluate post-NAC ALN status, and different algorithms were used respectively. For clinical model, univariate analysis was used to select the risk factors and N stage, long axis, short axis, cortical thickness of ALN, ER, and Ki-67 were identified as independent risk factors. Then backward-stepwise regression was used to build the clinical model with the AUC of 0.851 in external test cohort which was consistent with those of another research. Wang et al. [28] built a clinical model to predict ALN status after NAC with 320 breast cancer patients included, and the AUC value was 0.802. Kim et al. [22] developed a clinical model based on 408 women from one medical institution to predict the ALN response to NAC in node-positive breast cancer patients using MRI and ultrasound, and the AUC value in the validation sets reached 0.78. The small difference between AUC value of clinical models in different studies maybe due to the discrepancy of enrolled and selected the clinical factors. For radiomics model, MRMR and LASSO regression were applied, and nine most valuable radiomics features were selected and put into the model. The AUC value was 0.761 in validation cohort and 0.723 in external test cohort indicating that radiomic model based on AUS is predictive but not reliable. We speculated that morphologic changes in ALN caused by response to NAC might also hinder radiomics model to correctly recognize ALN metastases after NAC. More research could be carried out to overcome this problem. In addition, we also developed RNWCF, an integrated model combining clinical characteristics (N stage, long axis, short axis, cortical thickness of the ALN, ER, Ki-67) and radiomics signatures based on the multivariable logistic regression and had an AUC of 0.858 in the external test cohort. Currently, most studies focused on radiomic models or integrated models involved radiomics features for the prediction of preoperative ALN status in initially diagnosed breast cancer [17, 23–25]. Only a few studies have attempted to explore the effective approach involved radiomic signatures to predict ALN status after NAC for breast cancer patients. Zhou et al. [4] determined the feasibility of an integrated model containing radiomic signatures which derived from machine learning for the prediction of ALN pCR after NAC based on 247 patients from two institutions and the AUC value was 0.85 in validation cohort. However, they did not show the single predictive value of radiomic approach for ALN residue disease prediction. In addition, although those predictive models for ALN status evaluation, before or after NAC, achieved fine prediction performance in training and internal validation cohort, they did not have an external test set to investigate the generalizability [17, 23–25].

Unsatisfied generalizability in external test would hinder the translation of radiomics model into clinical practice, and the gap between radiomics model research and clinical practice should be filled up. Therefore, the heterogeneity between different ultrasound machines and different ultrasound protocols must be given more attention to. A Z-score normalization approach was applied to ameliorate this problem in our study, and the two-center research protocol and results (patients from 2 institutions and ALNU images from 4 ultrasound machines) proved the reliability of our model.

Consistent with previous studies, N stage and Ki-67 level were selected as independent risk factors and incorporated into the RNWCF in the present study. As an immunohistochemical proliferation marker, Ki67 has been extensively studied and explored to evaluate the ALN response to NAC in breast cancer [26]. It has already been reported that Ki-67 levels indicate proliferating cell levels, so higher Ki-67 levels indicate a higher pCR rate [27]. Cortical thickness also helped to identify pCR in the clinical model and RNWCF. The increase in cortical thickness shown on ALNU was regarded as association with malignancy [28]. Tumor cell infiltration in ALNs could cause cortical thickening. Greater cortical thickness was more likely to be related to a poor response to NAC and therefore less likely to achieve pCR [29].

This study had several limitations. First, selective bias and inherent errors were inevitable in a retrospective study. Second, though our study had a relatively large sample size than those from previous studies, multicenter analyses with a larger sample are required in the future. Finally, all ROIs in this study were drawn manually thus discrepancy between operator was inevitable. Therefore, we plan to conduct automatic segmentation and feature selection to optimize the construction of prediction models in future studies.

## Conclusion

In conclusion, we developed the clinical model, radiomics model and integrated model (RNWCF) for the prediction of ALN status after NAC. The RNWCF, combining the clinical characteristics and radiomics features of ALN, showed favorable predictive efficacy for ALN status evaluation. Therefore, the RNWCF could potentially serve as a noninvasive approach to assess the response of ALN to NAC, assisting personalized treatment strategies making, guiding ALN management, and probably avoiding unnecessary ALND. Further studies such as deep learning and prospective studies would be carried out, which could avoid the manual segmentation and determine the clinical feasibility of our predictive model.

## Supplementary Information

**Additional file 1.** Univariate analysis and radiomic features.

## Data Availability

The datasets used and/or analyzed during the current study are available from the corresponding author on reasonable request.

## Abbreviations

ALN: Axillary lymph node
ALND: Axillary lymph node dissection
NAC: Neoadjuvant chemotherapy
pCR: Pathological complete response
MRI: Magnetic resonance imaging
ALNU: Axillary lymph node ultrasound
ML: Machine learning
ER: Estrogen receptor
HER2: Human epidermal growth factor receptor 2
OR: Odds ratios
CI: Confidence intervals
ROI: Region of interest
GLCM: Gray level co-occurrence matrix
GLDM: Gray level dependence matrix
GLRLM: Gray level run length matrix
GLSZM: Gray level size zone matrix
MRMR: Minimal redundancy maximum relevance
LASSO: Least absolute shrinkage and selection operator
RNWCF: Radiomics nomogram with clinical factors
ROC: Receiver operating characteristic
AUC: Area under the receiver operating characteristic curve
