# Supplementary material for: Noninvasive prediction of node-positive breast cancer response to presurgical neoadjuvant chemotherapy therapy based on machine learning of axillary lymph node ultrasound
Source: J Transl Med. 2023 May 21;21:337. doi: 10.1186/s12967-023-04201-8 (PMC10201761; doi:10.1186/s12967-023-04201-8)
Supplement: Supplementary file 1 — Additional file 1. Univariate analysis and radiomic features. [file 12967_2023_4201_MOESM1_ESM.docx]

**1. The details of NAC regimen and course**

T: taxane (include docetaxel, albumin paclitaxel, and paclitaxel),

A: anthracycline (include epirubicin, pirarubicin and doxorubicin)

C: cyclophosphamide

H: trastuzumab

P: pertuzumab

Cb: carboplatin

TAC: taxane plus anthracycline and cyclophosphamide every 3 weeks for 6 cycles.

AC-T: anthracycline plus cyclophosphamide every 3 weeks for 4 cycles followed by taxane every 3 weeks for 4 cycles.

AT: anthracycline plus taxane every 3 weeks for 6 cycles.

AC-TH: anthracycline plus cyclophosphamide every 3 weeks for 4 cycles followed by taxane plus trastuzumab every 3 weeks for 4 cycles.

AC-THP: anthracycline plus cyclophosphamide every 3 weeks for 4 cycles followed by taxane plus trastuzumab and pertuzumab every 3 weeks for 4 cycles.

TH: taxane plus trastuzumab every 3 weeks for 6 cycles.

THP: taxane plus trastuzumab and pertuzumab every 3 weeks for 6 cycles.

TCb: taxane plus carboplatin every 3 weeks for 6 cycles.

TCbH: taxane plus carboplatin and trastuzumab every 3 weeks for 6 cycles.

TCbHP: taxane and carboplatin plus trastuzumab and pertuzumab every 3 weeks for 6 cycles.

**2. The detail of radiomic features**.

original_shape2D_Elongation

original_shape2D_MajorAxisLength

original_shape2D_MaximumDiameter

original_shape2D_MeshSurface

original_shape2D_MinorAxisLength

original_shape2D_Perimeter

original_shape2D_PerimeterSurfaceRatio

original_shape2D_PixelSurface

original_shape2D_Sphericity

lbp-2D_firstorder_10Percentile

lbp-2D_firstorder_90Percentile

lbp-2D_firstorder_Energy

lbp-2D_firstorder_Entropy

lbp-2D_firstorder_InterquartileRange

lbp-2D_firstorder_Kurtosis

lbp-2D_firstorder_Maximum

lbp-2D_firstorder_MeanAbsoluteDeviation

lbp-2D_firstorder_Mean

lbp-2D_firstorder_Median

lbp-2D_firstorder_Minimum

lbp-2D_firstorder_Range

lbp-2D_firstorder_RobustMeanAbsoluteDeviation

lbp-2D_firstorder_RootMeanSquared

lbp-2D_firstorder_Skewness

lbp-2D_firstorder_TotalEnergy

lbp-2D_firstorder_Uniformity

lbp-2D_firstorder_Variance

lbp-2D_glcm_Autocorrelation

lbp-2D_glcm_ClusterProminence

lbp-2D_glcm_ClusterShade

lbp-2D_glcm_ClusterTendency

lbp-2D_glcm_Contrast

lbp-2D_glcm_Correlation

lbp-2D_glcm_DifferenceAverage

lbp-2D_glcm_DifferenceEntropy

lbp-2D_glcm_DifferenceVariance

lbp-2D_glcm_Id

lbp-2D_glcm_Idm

lbp-2D_glcm_Idmn

lbp-2D_glcm_Idn

lbp-2D_glcm_Imc1

lbp-2D_glcm_Imc2

lbp-2D_glcm_InverseVariance

lbp-2D_glcm_JointAverage

lbp-2D_glcm_JointEnergy

lbp-2D_glcm_JointEntropy

lbp-2D_glcm_MCC

lbp-2D_glcm_MaximumProbability

lbp-2D_glcm_SumAverage

lbp-2D_glcm_SumEntropy

lbp-2D_glcm_SumSquares

lbp-2D_gldm_DependenceEntropy

lbp-2D_gldm_DependenceNonUniformity

lbp-2D_gldm_DependenceNonUniformityNormalized

lbp-2D_gldm_DependenceVariance

lbp-2D_gldm_GrayLevelNonUniformity

lbp-2D_gldm_GrayLevelVariance

lbp-2D_gldm_HighGrayLevelEmphasis

lbp-2D_gldm_LargeDependenceEmphasis

lbp-2D_gldm_LargeDependenceHighGrayLevelEmphasis

lbp-2D_gldm_LargeDependenceLowGrayLevelEmphasis

lbp-2D_gldm_LowGrayLevelEmphasis

lbp-2D_gldm_SmallDependenceEmphasis

lbp-2D_gldm_SmallDependenceHighGrayLevelEmphasis

lbp-2D_gldm_SmallDependenceLowGrayLevelEmphasis

lbp-2D_glrlm_GrayLevelNonUniformity

lbp-2D_glrlm_GrayLevelNonUniformityNormalized

lbp-2D_glrlm_GrayLevelVariance

lbp-2D_glrlm_HighGrayLevelRunEmphasis

lbp-2D_glrlm_LongRunEmphasis

lbp-2D_glrlm_LongRunHighGrayLevelEmphasis

lbp-2D_glrlm_LongRunLowGrayLevelEmphasis

lbp-2D_glrlm_LowGrayLevelRunEmphasis

lbp-2D_glrlm_RunEntropy

lbp-2D_glrlm_RunLengthNonUniformity

lbp-2D_glrlm_RunLengthNonUniformityNormalized

lbp-2D_glrlm_RunPercentage

lbp-2D_glrlm_RunVariance

lbp-2D_glrlm_ShortRunEmphasis

lbp-2D_glrlm_ShortRunHighGrayLevelEmphasis

lbp-2D_glrlm_ShortRunLowGrayLevelEmphasis

lbp-2D_glszm_GrayLevelNonUniformity

lbp-2D_glszm_GrayLevelNonUniformityNormalized

lbp-2D_glszm_GrayLevelVariance

lbp-2D_glszm_HighGrayLevelZoneEmphasis

lbp-2D_glszm_LargeAreaEmphasis

lbp-2D_glszm_LargeAreaHighGrayLevelEmphasis

lbp-2D_glszm_LargeAreaLowGrayLevelEmphasis

lbp-2D_glszm_LowGrayLevelZoneEmphasis

lbp-2D_glszm_SizeZoneNonUniformity

lbp-2D_glszm_SizeZoneNonUniformityNormalized

lbp-2D_glszm_SmallAreaEmphasis

lbp-2D_glszm_SmallAreaHighGrayLevelEmphasis

lbp-2D_glszm_SmallAreaLowGrayLevelEmphasis

lbp-2D_glszm_ZoneEntropy

lbp-2D_glszm_ZonePercentage

lbp-2D_glszm_ZoneVariance

lbp-2D_ngtdm_Busyness

lbp-2D_ngtdm_Coarseness

lbp-2D_ngtdm_Complexity

lbp-2D_ngtdm_Contrast

lbp-2D_ngtdm_Strength

log-sigma-0-1-mm-3D_firstorder_10Percentile

log-sigma-0-1-mm-3D_firstorder_90Percentile

log-sigma-0-1-mm-3D_firstorder_Energy

log-sigma-0-1-mm-3D_firstorder_Entropy

log-sigma-0-1-mm-3D_firstorder_InterquartileRange

log-sigma-0-1-mm-3D_firstorder_Kurtosis

log-sigma-0-1-mm-3D_firstorder_Maximum

log-sigma-0-1-mm-3D_firstorder_MeanAbsoluteDeviation

log-sigma-0-1-mm-3D_firstorder_Mean

log-sigma-0-1-mm-3D_firstorder_Median

log-sigma-0-1-mm-3D_firstorder_Minimum

log-sigma-0-1-mm-3D_firstorder_Range

log-sigma-0-1-mm-3D_firstorder_RobustMeanAbsoluteDeviation

log-sigma-0-1-mm-3D_firstorder_RootMeanSquared

log-sigma-0-1-mm-3D_firstorder_Skewness

log-sigma-0-1-mm-3D_firstorder_TotalEnergy

log-sigma-0-1-mm-3D_firstorder_Uniformity

log-sigma-0-1-mm-3D_firstorder_Variance

log-sigma-0-1-mm-3D_glcm_Autocorrelation

log-sigma-0-1-mm-3D_glcm_ClusterProminence

log-sigma-0-1-mm-3D_glcm_ClusterShade

log-sigma-0-1-mm-3D_glcm_ClusterTendency

log-sigma-0-1-mm-3D_glcm_Contrast

log-sigma-0-1-mm-3D_glcm_Correlation

log-sigma-0-1-mm-3D_glcm_DifferenceAverage

log-sigma-0-1-mm-3D_glcm_DifferenceEntropy

log-sigma-0-1-mm-3D_glcm_DifferenceVariance

log-sigma-0-1-mm-3D_glcm_Id

log-sigma-0-1-mm-3D_glcm_Idm

log-sigma-0-1-mm-3D_glcm_Idmn

log-sigma-0-1-mm-3D_glcm_Idn

log-sigma-0-1-mm-3D_glcm_Imc1

log-sigma-0-1-mm-3D_glcm_Imc2

log-sigma-0-1-mm-3D_glcm_InverseVariance

log-sigma-0-1-mm-3D_glcm_JointAverage

log-sigma-0-1-mm-3D_glcm_JointEnergy

log-sigma-0-1-mm-3D_glcm_JointEntropy

log-sigma-0-1-mm-3D_glcm_MCC

log-sigma-0-1-mm-3D_glcm_MaximumProbability

log-sigma-0-1-mm-3D_glcm_SumAverage

log-sigma-0-1-mm-3D_glcm_SumEntropy

log-sigma-0-1-mm-3D_glcm_SumSquares

log-sigma-0-1-mm-3D_gldm_DependenceEntropy

log-sigma-0-1-mm-3D_gldm_DependenceNonUniformity

log-sigma-0-1-mm-3D_gldm_DependenceNonUniformityNormalized

log-sigma-0-1-mm-3D_gldm_DependenceVariance

log-sigma-0-1-mm-3D_gldm_GrayLevelNonUniformity

log-sigma-0-1-mm-3D_gldm_GrayLevelVariance

log-sigma-0-1-mm-3D_gldm_HighGrayLevelEmphasis

log-sigma-0-1-mm-3D_gldm_LargeDependenceEmphasis

log-sigma-0-1-mm-3D_gldm_LargeDependenceHighGrayLevelEmphasis

log-sigma-0-1-mm-3D_gldm_LargeDependenceLowGrayLevelEmphasis

log-sigma-0-1-mm-3D_gldm_LowGrayLevelEmphasis

log-sigma-0-1-mm-3D_gldm_SmallDependenceEmphasis

log-sigma-0-1-mm-3D_gldm_SmallDependenceHighGrayLevelEmphasis

log-sigma-0-1-mm-3D_gldm_SmallDependenceLowGrayLevelEmphasis

log-sigma-0-1-mm-3D_glrlm_GrayLevelNonUniformity

log-sigma-0-1-mm-3D_glrlm_GrayLevelNonUniformityNormalized

log-sigma-0-1-mm-3D_glrlm_GrayLevelVariance

log-sigma-0-1-mm-3D_glrlm_HighGrayLevelRunEmphasis

log-sigma-0-1-mm-3D_glrlm_LongRunEmphasis

log-sigma-0-1-mm-3D_glrlm_LongRunHighGrayLevelEmphasis

log-sigma-0-1-mm-3D_glrlm_LongRunLowGrayLevelEmphasis

log-sigma-0-1-mm-3D_glrlm_LowGrayLevelRunEmphasis

log-sigma-0-1-mm-3D_glrlm_RunEntropy

log-sigma-0-1-mm-3D_glrlm_RunLengthNonUniformity

log-sigma-0-1-mm-3D_glrlm_RunLengthNonUniformityNormalized

log-sigma-0-1-mm-3D_glrlm_RunPercentage

log-sigma-0-1-mm-3D_glrlm_RunVariance

log-sigma-0-1-mm-3D_glrlm_ShortRunEmphasis

log-sigma-0-1-mm-3D_glrlm_ShortRunHighGrayLevelEmphasis

log-sigma-0-1-mm-3D_glrlm_ShortRunLowGrayLevelEmphasis

log-sigma-0-1-mm-3D_glszm_GrayLevelNonUniformity

log-sigma-0-1-mm-3D_glszm_GrayLevelNonUniformityNormalized

log-sigma-0-1-mm-3D_glszm_GrayLevelVariance

log-sigma-0-1-mm-3D_glszm_HighGrayLevelZoneEmphasis

log-sigma-0-1-mm-3D_glszm_LargeAreaEmphasis

log-sigma-0-1-mm-3D_glszm_LargeAreaHighGrayLevelEmphasis

log-sigma-0-1-mm-3D_glszm_LargeAreaLowGrayLevelEmphasis

log-sigma-0-1-mm-3D_glszm_LowGrayLevelZoneEmphasis

log-sigma-0-1-mm-3D_glszm_SizeZoneNonUniformity

log-sigma-0-1-mm-3D_glszm_SizeZoneNonUniformityNormalized

log-sigma-0-1-mm-3D_glszm_SmallAreaEmphasis

log-sigma-0-1-mm-3D_glszm_SmallAreaHighGrayLevelEmphasis

log-sigma-0-1-mm-3D_glszm_SmallAreaLowGrayLevelEmphasis

log-sigma-0-1-mm-3D_glszm_ZoneEntropy

log-sigma-0-1-mm-3D_glszm_ZonePercentage

log-sigma-0-1-mm-3D_glszm_ZoneVariance

log-sigma-0-1-mm-3D_ngtdm_Busyness

log-sigma-0-1-mm-3D_ngtdm_Coarseness

log-sigma-0-1-mm-3D_ngtdm_Complexity

log-sigma-0-1-mm-3D_ngtdm_Contrast

log-sigma-0-1-mm-3D_ngtdm_Strength

log-sigma-0-2-mm-3D_firstorder_10Percentile

log-sigma-0-2-mm-3D_firstorder_90Percentile

log-sigma-0-2-mm-3D_firstorder_Energy

log-sigma-0-2-mm-3D_firstorder_Entropy

log-sigma-0-2-mm-3D_firstorder_InterquartileRange

log-sigma-0-2-mm-3D_firstorder_Kurtosis

log-sigma-0-2-mm-3D_firstorder_Maximum

log-sigma-0-2-mm-3D_firstorder_MeanAbsoluteDeviation

log-sigma-0-2-mm-3D_firstorder_Mean

log-sigma-0-2-mm-3D_firstorder_Median

log-sigma-0-2-mm-3D_firstorder_Minimum

log-sigma-0-2-mm-3D_firstorder_Range

log-sigma-0-2-mm-3D_firstorder_RobustMeanAbsoluteDeviation

log-sigma-0-2-mm-3D_firstorder_RootMeanSquared

log-sigma-0-2-mm-3D_firstorder_Skewness

log-sigma-0-2-mm-3D_firstorder_TotalEnergy

log-sigma-0-2-mm-3D_firstorder_Uniformity

log-sigma-0-2-mm-3D_firstorder_Variance

log-sigma-0-2-mm-3D_glcm_Autocorrelation

log-sigma-0-2-mm-3D_glcm_ClusterProminence

log-sigma-0-2-mm-3D_glcm_ClusterShade

log-sigma-0-2-mm-3D_glcm_ClusterTendency

log-sigma-0-2-mm-3D_glcm_Contrast

log-sigma-0-2-mm-3D_glcm_Correlation

log-sigma-0-2-mm-3D_glcm_DifferenceAverage

log-sigma-0-2-mm-3D_glcm_DifferenceEntropy

log-sigma-0-2-mm-3D_glcm_DifferenceVariance

log-sigma-0-2-mm-3D_glcm_Id

log-sigma-0-2-mm-3D_glcm_Idm

log-sigma-0-2-mm-3D_glcm_Idmn

log-sigma-0-2-mm-3D_glcm_Idn

log-sigma-0-2-mm-3D_glcm_Imc1

log-sigma-0-2-mm-3D_glcm_Imc2

log-sigma-0-2-mm-3D_glcm_InverseVariance

log-sigma-0-2-mm-3D_glcm_JointAverage

log-sigma-0-2-mm-3D_glcm_JointEnergy

log-sigma-0-2-mm-3D_glcm_JointEntropy

log-sigma-0-2-mm-3D_glcm_MCC

log-sigma-0-2-mm-3D_glcm_MaximumProbability

log-sigma-0-2-mm-3D_glcm_SumAverage

log-sigma-0-2-mm-3D_glcm_SumEntropy

log-sigma-0-2-mm-3D_glcm_SumSquares

log-sigma-0-2-mm-3D_gldm_DependenceEntropy

log-sigma-0-2-mm-3D_gldm_DependenceNonUniformity

log-sigma-0-2-mm-3D_gldm_DependenceNonUniformityNormalized

log-sigma-0-2-mm-3D_gldm_DependenceVariance

log-sigma-0-2-mm-3D_gldm_GrayLevelNonUniformity

log-sigma-0-2-mm-3D_gldm_GrayLevelVariance

log-sigma-0-2-mm-3D_gldm_HighGrayLevelEmphasis

log-sigma-0-2-mm-3D_gldm_LargeDependenceEmphasis

log-sigma-0-2-mm-3D_gldm_LargeDependenceHighGrayLevelEmphasis

log-sigma-0-2-mm-3D_gldm_LargeDependenceLowGrayLevelEmphasis

log-sigma-0-2-mm-3D_gldm_LowGrayLevelEmphasis

log-sigma-0-2-mm-3D_gldm_SmallDependenceEmphasis

log-sigma-0-2-mm-3D_gldm_SmallDependenceHighGrayLevelEmphasis

log-sigma-0-2-mm-3D_gldm_SmallDependenceLowGrayLevelEmphasis

log-sigma-0-2-mm-3D_glrlm_GrayLevelNonUniformity

log-sigma-0-2-mm-3D_glrlm_GrayLevelNonUniformityNormalized

log-sigma-0-2-mm-3D_glrlm_GrayLevelVariance

log-sigma-0-2-mm-3D_glrlm_HighGrayLevelRunEmphasis

log-sigma-0-2-mm-3D_glrlm_LongRunEmphasis

log-sigma-0-2-mm-3D_glrlm_LongRunHighGrayLevelEmphasis

log-sigma-0-2-mm-3D_glrlm_LongRunLowGrayLevelEmphasis

log-sigma-0-2-mm-3D_glrlm_LowGrayLevelRunEmphasis

log-sigma-0-2-mm-3D_glrlm_RunEntropy

log-sigma-0-2-mm-3D_glrlm_RunLengthNonUniformity

log-sigma-0-2-mm-3D_glrlm_RunLengthNonUniformityNormalized

log-sigma-0-2-mm-3D_glrlm_RunPercentage

log-sigma-0-2-mm-3D_glrlm_RunVariance

log-sigma-0-2-mm-3D_glrlm_ShortRunEmphasis

log-sigma-0-2-mm-3D_glrlm_ShortRunHighGrayLevelEmphasis

log-sigma-0-2-mm-3D_glrlm_ShortRunLowGrayLevelEmphasis

log-sigma-0-2-mm-3D_glszm_GrayLevelNonUniformity

log-sigma-0-2-mm-3D_glszm_GrayLevelNonUniformityNormalized

log-sigma-0-2-mm-3D_glszm_GrayLevelVariance

log-sigma-0-2-mm-3D_glszm_HighGrayLevelZoneEmphasis

log-sigma-0-2-mm-3D_glszm_LargeAreaEmphasis

log-sigma-0-2-mm-3D_glszm_LargeAreaHighGrayLevelEmphasis

log-sigma-0-2-mm-3D_glszm_LargeAreaLowGrayLevelEmphasis

log-sigma-0-2-mm-3D_glszm_LowGrayLevelZoneEmphasis

log-sigma-0-2-mm-3D_glszm_SizeZoneNonUniformity

log-sigma-0-2-mm-3D_glszm_SizeZoneNonUniformityNormalized

log-sigma-0-2-mm-3D_glszm_SmallAreaEmphasis

log-sigma-0-2-mm-3D_glszm_SmallAreaHighGrayLevelEmphasis

log-sigma-0-2-mm-3D_glszm_SmallAreaLowGrayLevelEmphasis

log-sigma-0-2-mm-3D_glszm_ZoneEntropy

log-sigma-0-2-mm-3D_glszm_ZonePercentage

log-sigma-0-2-mm-3D_glszm_ZoneVariance

log-sigma-0-2-mm-3D_ngtdm_Busyness

log-sigma-0-2-mm-3D_ngtdm_Coarseness

log-sigma-0-2-mm-3D_ngtdm_Complexity

log-sigma-0-2-mm-3D_ngtdm_Contrast

log-sigma-0-2-mm-3D_ngtdm_Strength

log-sigma-0-3-mm-3D_firstorder_10Percentile

log-sigma-0-3-mm-3D_firstorder_90Percentile

log-sigma-0-3-mm-3D_firstorder_Energy

log-sigma-0-3-mm-3D_firstorder_Entropy

log-sigma-0-3-mm-3D_firstorder_InterquartileRange

log-sigma-0-3-mm-3D_firstorder_Kurtosis

log-sigma-0-3-mm-3D_firstorder_Maximum

log-sigma-0-3-mm-3D_firstorder_MeanAbsoluteDeviation

log-sigma-0-3-mm-3D_firstorder_Mean

log-sigma-0-3-mm-3D_firstorder_Median

log-sigma-0-3-mm-3D_firstorder_Minimum

log-sigma-0-3-mm-3D_firstorder_Range

log-sigma-0-3-mm-3D_firstorder_RobustMeanAbsoluteDeviation

log-sigma-0-3-mm-3D_firstorder_RootMeanSquared

log-sigma-0-3-mm-3D_firstorder_Skewness

log-sigma-0-3-mm-3D_firstorder_TotalEnergy

log-sigma-0-3-mm-3D_firstorder_Uniformity

log-sigma-0-3-mm-3D_firstorder_Variance

log-sigma-0-3-mm-3D_glcm_Autocorrelation

log-sigma-0-3-mm-3D_glcm_ClusterProminence

log-sigma-0-3-mm-3D_glcm_ClusterShade

log-sigma-0-3-mm-3D_glcm_ClusterTendency

log-sigma-0-3-mm-3D_glcm_Contrast

log-sigma-0-3-mm-3D_glcm_Correlation

log-sigma-0-3-mm-3D_glcm_DifferenceAverage

log-sigma-0-3-mm-3D_glcm_DifferenceEntropy

log-sigma-0-3-mm-3D_glcm_DifferenceVariance

log-sigma-0-3-mm-3D_glcm_Id

log-sigma-0-3-mm-3D_glcm_Idm

log-sigma-0-3-mm-3D_glcm_Idmn

log-sigma-0-3-mm-3D_glcm_Idn

log-sigma-0-3-mm-3D_glcm_Imc1

log-sigma-0-3-mm-3D_glcm_Imc2

log-sigma-0-3-mm-3D_glcm_InverseVariance

log-sigma-0-3-mm-3D_glcm_JointAverage

log-sigma-0-3-mm-3D_glcm_JointEnergy

log-sigma-0-3-mm-3D_glcm_JointEntropy

log-sigma-0-3-mm-3D_glcm_MCC

log-sigma-0-3-mm-3D_glcm_MaximumProbability

log-sigma-0-3-mm-3D_glcm_SumAverage

log-sigma-0-3-mm-3D_glcm_SumEntropy

log-sigma-0-3-mm-3D_glcm_SumSquares

log-sigma-0-3-mm-3D_gldm_DependenceEntropy

log-sigma-0-3-mm-3D_gldm_DependenceNonUniformity

log-sigma-0-3-mm-3D_gldm_DependenceNonUniformityNormalized

log-sigma-0-3-mm-3D_gldm_DependenceVariance

log-sigma-0-3-mm-3D_gldm_GrayLevelNonUniformity

log-sigma-0-3-mm-3D_gldm_GrayLevelVariance

log-sigma-0-3-mm-3D_gldm_HighGrayLevelEmphasis

log-sigma-0-3-mm-3D_gldm_LargeDependenceEmphasis

log-sigma-0-3-mm-3D_gldm_LargeDependenceHighGrayLevelEmphasis

log-sigma-0-3-mm-3D_gldm_LargeDependenceLowGrayLevelEmphasis

log-sigma-0-3-mm-3D_gldm_LowGrayLevelEmphasis

log-sigma-0-3-mm-3D_gldm_SmallDependenceEmphasis

log-sigma-0-3-mm-3D_gldm_SmallDependenceHighGrayLevelEmphasis

log-sigma-0-3-mm-3D_gldm_SmallDependenceLowGrayLevelEmphasis

log-sigma-0-3-mm-3D_glrlm_GrayLevelNonUniformity

log-sigma-0-3-mm-3D_glrlm_GrayLevelNonUniformityNormalized

log-sigma-0-3-mm-3D_glrlm_GrayLevelVariance

log-sigma-0-3-mm-3D_glrlm_HighGrayLevelRunEmphasis

log-sigma-0-3-mm-3D_glrlm_LongRunEmphasis

log-sigma-0-3-mm-3D_glrlm_LongRunHighGrayLevelEmphasis

log-sigma-0-3-mm-3D_glrlm_LongRunLowGrayLevelEmphasis

log-sigma-0-3-mm-3D_glrlm_LowGrayLevelRunEmphasis

log-sigma-0-3-mm-3D_glrlm_RunEntropy

log-sigma-0-3-mm-3D_glrlm_RunLengthNonUniformity

log-sigma-0-3-mm-3D_glrlm_RunLengthNonUniformityNormalized

log-sigma-0-3-mm-3D_glrlm_RunPercentage

log-sigma-0-3-mm-3D_glrlm_RunVariance

log-sigma-0-3-mm-3D_glrlm_ShortRunEmphasis

log-sigma-0-3-mm-3D_glrlm_ShortRunHighGrayLevelEmphasis

log-sigma-0-3-mm-3D_glrlm_ShortRunLowGrayLevelEmphasis

log-sigma-0-3-mm-3D_glszm_GrayLevelNonUniformity

log-sigma-0-3-mm-3D_glszm_GrayLevelNonUniformityNormalized

log-sigma-0-3-mm-3D_glszm_GrayLevelVariance

log-sigma-0-3-mm-3D_glszm_HighGrayLevelZoneEmphasis

log-sigma-0-3-mm-3D_glszm_LargeAreaEmphasis

log-sigma-0-3-mm-3D_glszm_LargeAreaHighGrayLevelEmphasis

log-sigma-0-3-mm-3D_glszm_LargeAreaLowGrayLevelEmphasis

log-sigma-0-3-mm-3D_glszm_LowGrayLevelZoneEmphasis

log-sigma-0-3-mm-3D_glszm_SizeZoneNonUniformity

log-sigma-0-3-mm-3D_glszm_SizeZoneNonUniformityNormalized

log-sigma-0-3-mm-3D_glszm_SmallAreaEmphasis

log-sigma-0-3-mm-3D_glszm_SmallAreaHighGrayLevelEmphasis

log-sigma-0-3-mm-3D_glszm_SmallAreaLowGrayLevelEmphasis

log-sigma-0-3-mm-3D_glszm_ZoneEntropy

log-sigma-0-3-mm-3D_glszm_ZonePercentage

log-sigma-0-3-mm-3D_glszm_ZoneVariance

log-sigma-0-3-mm-3D_ngtdm_Busyness

log-sigma-0-3-mm-3D_ngtdm_Coarseness

log-sigma-0-3-mm-3D_ngtdm_Complexity

log-sigma-0-3-mm-3D_ngtdm_Contrast

log-sigma-0-3-mm-3D_ngtdm_Strength

log-sigma-0-4-mm-3D_firstorder_10Percentile

log-sigma-0-4-mm-3D_firstorder_90Percentile

log-sigma-0-4-mm-3D_firstorder_Energy

log-sigma-0-4-mm-3D_firstorder_Entropy

log-sigma-0-4-mm-3D_firstorder_InterquartileRange

log-sigma-0-4-mm-3D_firstorder_Kurtosis

log-sigma-0-4-mm-3D_firstorder_Maximum

log-sigma-0-4-mm-3D_firstorder_MeanAbsoluteDeviation

log-sigma-0-4-mm-3D_firstorder_Mean

log-sigma-0-4-mm-3D_firstorder_Median

log-sigma-0-4-mm-3D_firstorder_Minimum

log-sigma-0-4-mm-3D_firstorder_Range

log-sigma-0-4-mm-3D_firstorder_RobustMeanAbsoluteDeviation

log-sigma-0-4-mm-3D_firstorder_RootMeanSquared

log-sigma-0-4-mm-3D_firstorder_Skewness

log-sigma-0-4-mm-3D_firstorder_TotalEnergy

log-sigma-0-4-mm-3D_firstorder_Uniformity

log-sigma-0-4-mm-3D_firstorder_Variance

log-sigma-0-4-mm-3D_glcm_Autocorrelation

log-sigma-0-4-mm-3D_glcm_ClusterProminence

log-sigma-0-4-mm-3D_glcm_ClusterShade

log-sigma-0-4-mm-3D_glcm_ClusterTendency

log-sigma-0-4-mm-3D_glcm_Contrast

log-sigma-0-4-mm-3D_glcm_Correlation

log-sigma-0-4-mm-3D_glcm_DifferenceAverage

log-sigma-0-4-mm-3D_glcm_DifferenceEntropy

log-sigma-0-4-mm-3D_glcm_DifferenceVariance

log-sigma-0-4-mm-3D_glcm_Id

log-sigma-0-4-mm-3D_glcm_Idm

log-sigma-0-4-mm-3D_glcm_Idmn

log-sigma-0-4-mm-3D_glcm_Idn

log-sigma-0-4-mm-3D_glcm_Imc1

log-sigma-0-4-mm-3D_glcm_Imc2

log-sigma-0-4-mm-3D_glcm_InverseVariance

log-sigma-0-4-mm-3D_glcm_JointAverage

log-sigma-0-4-mm-3D_glcm_JointEnergy

log-sigma-0-4-mm-3D_glcm_JointEntropy

log-sigma-0-4-mm-3D_glcm_MCC

log-sigma-0-4-mm-3D_glcm_MaximumProbability

log-sigma-0-4-mm-3D_glcm_SumAverage

log-sigma-0-4-mm-3D_glcm_SumEntropy

log-sigma-0-4-mm-3D_glcm_SumSquares

log-sigma-0-4-mm-3D_gldm_DependenceEntropy

log-sigma-0-4-mm-3D_gldm_DependenceNonUniformity

log-sigma-0-4-mm-3D_gldm_DependenceNonUniformityNormalized

log-sigma-0-4-mm-3D_gldm_DependenceVariance

log-sigma-0-4-mm-3D_gldm_GrayLevelNonUniformity

log-sigma-0-4-mm-3D_gldm_GrayLevelVariance

log-sigma-0-4-mm-3D_gldm_HighGrayLevelEmphasis

log-sigma-0-4-mm-3D_gldm_LargeDependenceEmphasis

log-sigma-0-4-mm-3D_gldm_LargeDependenceHighGrayLevelEmphasis

log-sigma-0-4-mm-3D_gldm_LargeDependenceLowGrayLevelEmphasis

log-sigma-0-4-mm-3D_gldm_LowGrayLevelEmphasis

log-sigma-0-4-mm-3D_gldm_SmallDependenceEmphasis

log-sigma-0-4-mm-3D_gldm_SmallDependenceHighGrayLevelEmphasis

log-sigma-0-4-mm-3D_gldm_SmallDependenceLowGrayLevelEmphasis

log-sigma-0-4-mm-3D_glrlm_GrayLevelNonUniformity

log-sigma-0-4-mm-3D_glrlm_GrayLevelNonUniformityNormalized

log-sigma-0-4-mm-3D_glrlm_GrayLevelVariance

log-sigma-0-4-mm-3D_glrlm_HighGrayLevelRunEmphasis

log-sigma-0-4-mm-3D_glrlm_LongRunEmphasis

log-sigma-0-4-mm-3D_glrlm_LongRunHighGrayLevelEmphasis

log-sigma-0-4-mm-3D_glrlm_LongRunLowGrayLevelEmphasis

log-sigma-0-4-mm-3D_glrlm_LowGrayLevelRunEmphasis

log-sigma-0-4-mm-3D_glrlm_RunEntropy

log-sigma-0-4-mm-3D_glrlm_RunLengthNonUniformity

log-sigma-0-4-mm-3D_glrlm_RunLengthNonUniformityNormalized

log-sigma-0-4-mm-3D_glrlm_RunPercentage

log-sigma-0-4-mm-3D_glrlm_RunVariance

log-sigma-0-4-mm-3D_glrlm_ShortRunEmphasis

log-sigma-0-4-mm-3D_glrlm_ShortRunHighGrayLevelEmphasis

log-sigma-0-4-mm-3D_glrlm_ShortRunLowGrayLevelEmphasis

log-sigma-0-4-mm-3D_glszm_GrayLevelNonUniformity

log-sigma-0-4-mm-3D_glszm_GrayLevelNonUniformityNormalized

log-sigma-0-4-mm-3D_glszm_GrayLevelVariance

log-sigma-0-4-mm-3D_glszm_HighGrayLevelZoneEmphasis

log-sigma-0-4-mm-3D_glszm_LargeAreaEmphasis

log-sigma-0-4-mm-3D_glszm_LargeAreaHighGrayLevelEmphasis

log-sigma-0-4-mm-3D_glszm_LargeAreaLowGrayLevelEmphasis

log-sigma-0-4-mm-3D_glszm_LowGrayLevelZoneEmphasis

log-sigma-0-4-mm-3D_glszm_SizeZoneNonUniformity

log-sigma-0-4-mm-3D_glszm_SizeZoneNonUniformityNormalized

log-sigma-0-4-mm-3D_glszm_SmallAreaEmphasis

log-sigma-0-4-mm-3D_glszm_SmallAreaHighGrayLevelEmphasis

log-sigma-0-4-mm-3D_glszm_SmallAreaLowGrayLevelEmphasis

log-sigma-0-4-mm-3D_glszm_ZoneEntropy

log-sigma-0-4-mm-3D_glszm_ZonePercentage

log-sigma-0-4-mm-3D_glszm_ZoneVariance

log-sigma-0-4-mm-3D_ngtdm_Busyness

log-sigma-0-4-mm-3D_ngtdm_Coarseness

log-sigma-0-4-mm-3D_ngtdm_Complexity

log-sigma-0-4-mm-3D_ngtdm_Contrast

log-sigma-0-4-mm-3D_ngtdm_Strength

log-sigma-0-5-mm-3D_firstorder_10Percentile

log-sigma-0-5-mm-3D_firstorder_90Percentile

log-sigma-0-5-mm-3D_firstorder_Energy

log-sigma-0-5-mm-3D_firstorder_Entropy

log-sigma-0-5-mm-3D_firstorder_InterquartileRange

log-sigma-0-5-mm-3D_firstorder_Kurtosis

log-sigma-0-5-mm-3D_firstorder_Maximum

log-sigma-0-5-mm-3D_firstorder_MeanAbsoluteDeviation

log-sigma-0-5-mm-3D_firstorder_Mean

log-sigma-0-5-mm-3D_firstorder_Median

log-sigma-0-5-mm-3D_firstorder_Minimum

log-sigma-0-5-mm-3D_firstorder_Range

log-sigma-0-5-mm-3D_firstorder_RobustMeanAbsoluteDeviation

log-sigma-0-5-mm-3D_firstorder_RootMeanSquared

log-sigma-0-5-mm-3D_firstorder_Skewness

log-sigma-0-5-mm-3D_firstorder_TotalEnergy

log-sigma-0-5-mm-3D_firstorder_Uniformity

log-sigma-0-5-mm-3D_firstorder_Variance

log-sigma-0-5-mm-3D_glcm_Autocorrelation

log-sigma-0-5-mm-3D_glcm_ClusterProminence

log-sigma-0-5-mm-3D_glcm_ClusterShade

log-sigma-0-5-mm-3D_glcm_ClusterTendency

log-sigma-0-5-mm-3D_glcm_Contrast

log-sigma-0-5-mm-3D_glcm_Correlation

log-sigma-0-5-mm-3D_glcm_DifferenceAverage

log-sigma-0-5-mm-3D_glcm_DifferenceEntropy

log-sigma-0-5-mm-3D_glcm_DifferenceVariance

log-sigma-0-5-mm-3D_glcm_Id

log-sigma-0-5-mm-3D_glcm_Idm

log-sigma-0-5-mm-3D_glcm_Idmn

log-sigma-0-5-mm-3D_glcm_Idn

log-sigma-0-5-mm-3D_glcm_Imc1

log-sigma-0-5-mm-3D_glcm_Imc2

log-sigma-0-5-mm-3D_glcm_InverseVariance

log-sigma-0-5-mm-3D_glcm_JointAverage

log-sigma-0-5-mm-3D_glcm_JointEnergy

log-sigma-0-5-mm-3D_glcm_JointEntropy

log-sigma-0-5-mm-3D_glcm_MCC

log-sigma-0-5-mm-3D_glcm_MaximumProbability

log-sigma-0-5-mm-3D_glcm_SumAverage

log-sigma-0-5-mm-3D_glcm_SumEntropy

log-sigma-0-5-mm-3D_glcm_SumSquares

log-sigma-0-5-mm-3D_gldm_DependenceEntropy

log-sigma-0-5-mm-3D_gldm_DependenceNonUniformity

log-sigma-0-5-mm-3D_gldm_DependenceNonUniformityNormalized

log-sigma-0-5-mm-3D_gldm_DependenceVariance

log-sigma-0-5-mm-3D_gldm_GrayLevelNonUniformity

log-sigma-0-5-mm-3D_gldm_GrayLevelVariance

log-sigma-0-5-mm-3D_gldm_HighGrayLevelEmphasis

log-sigma-0-5-mm-3D_gldm_LargeDependenceEmphasis

log-sigma-0-5-mm-3D_gldm_LargeDependenceHighGrayLevelEmphasis

log-sigma-0-5-mm-3D_gldm_LargeDependenceLowGrayLevelEmphasis

log-sigma-0-5-mm-3D_gldm_LowGrayLevelEmphasis

log-sigma-0-5-mm-3D_gldm_SmallDependenceEmphasis

log-sigma-0-5-mm-3D_gldm_SmallDependenceHighGrayLevelEmphasis

log-sigma-0-5-mm-3D_gldm_SmallDependenceLowGrayLevelEmphasis

log-sigma-0-5-mm-3D_glrlm_GrayLevelNonUniformity

log-sigma-0-5-mm-3D_glrlm_GrayLevelNonUniformityNormalized

log-sigma-0-5-mm-3D_glrlm_GrayLevelVariance

log-sigma-0-5-mm-3D_glrlm_HighGrayLevelRunEmphasis

log-sigma-0-5-mm-3D_glrlm_LongRunEmphasis

log-sigma-0-5-mm-3D_glrlm_LongRunHighGrayLevelEmphasis

log-sigma-0-5-mm-3D_glrlm_LongRunLowGrayLevelEmphasis

log-sigma-0-5-mm-3D_glrlm_LowGrayLevelRunEmphasis

log-sigma-0-5-mm-3D_glrlm_RunEntropy

log-sigma-0-5-mm-3D_glrlm_RunLengthNonUniformity

log-sigma-0-5-mm-3D_glrlm_RunLengthNonUniformityNormalized

log-sigma-0-5-mm-3D_glrlm_RunPercentage

log-sigma-0-5-mm-3D_glrlm_RunVariance

log-sigma-0-5-mm-3D_glrlm_ShortRunEmphasis

log-sigma-0-5-mm-3D_glrlm_ShortRunHighGrayLevelEmphasis

log-sigma-0-5-mm-3D_glrlm_ShortRunLowGrayLevelEmphasis

log-sigma-0-5-mm-3D_glszm_GrayLevelNonUniformity

log-sigma-0-5-mm-3D_glszm_GrayLevelNonUniformityNormalized

log-sigma-0-5-mm-3D_glszm_GrayLevelVariance

log-sigma-0-5-mm-3D_glszm_HighGrayLevelZoneEmphasis

log-sigma-0-5-mm-3D_glszm_LargeAreaEmphasis

log-sigma-0-5-mm-3D_glszm_LargeAreaHighGrayLevelEmphasis

log-sigma-0-5-mm-3D_glszm_LargeAreaLowGrayLevelEmphasis

log-sigma-0-5-mm-3D_glszm_LowGrayLevelZoneEmphasis

log-sigma-0-5-mm-3D_glszm_SizeZoneNonUniformity

log-sigma-0-5-mm-3D_glszm_SizeZoneNonUniformityNormalized

log-sigma-0-5-mm-3D_glszm_SmallAreaEmphasis

log-sigma-0-5-mm-3D_glszm_SmallAreaHighGrayLevelEmphasis

log-sigma-0-5-mm-3D_glszm_SmallAreaLowGrayLevelEmphasis

log-sigma-0-5-mm-3D_glszm_ZoneEntropy

log-sigma-0-5-mm-3D_glszm_ZonePercentage

log-sigma-0-5-mm-3D_glszm_ZoneVariance

log-sigma-0-5-mm-3D_ngtdm_Busyness

log-sigma-0-5-mm-3D_ngtdm_Coarseness

log-sigma-0-5-mm-3D_ngtdm_Complexity

log-sigma-0-5-mm-3D_ngtdm_Contrast

log-sigma-0-5-mm-3D_ngtdm_Strength

original_firstorder_10Percentile

original_firstorder_90Percentile

original_firstorder_Energy

original_firstorder_Entropy

original_firstorder_InterquartileRange

original_firstorder_Kurtosis

original_firstorder_Maximum

original_firstorder_MeanAbsoluteDeviation

original_firstorder_Mean

original_firstorder_Median

original_firstorder_Minimum

original_firstorder_Range

original_firstorder_RobustMeanAbsoluteDeviation

original_firstorder_RootMeanSquared

original_firstorder_Skewness

original_firstorder_TotalEnergy

original_firstorder_Uniformity

original_firstorder_Variance

original_glcm_Autocorrelation

original_glcm_ClusterProminence

original_glcm_ClusterShade

original_glcm_ClusterTendency

original_glcm_Contrast

original_glcm_Correlation

original_glcm_DifferenceAverage

original_glcm_DifferenceEntropy

original_glcm_DifferenceVariance

original_glcm_Id

original_glcm_Idm

original_glcm_Idmn

original_glcm_Idn

original_glcm_Imc1

original_glcm_Imc2

original_glcm_InverseVariance

original_glcm_JointAverage

original_glcm_JointEnergy

original_glcm_JointEntropy

original_glcm_MCC

original_glcm_MaximumProbability

original_glcm_SumAverage

original_glcm_SumEntropy

original_glcm_SumSquares

original_gldm_DependenceEntropy

original_gldm_DependenceNonUniformity

original_gldm_DependenceNonUniformityNormalized

original_gldm_DependenceVariance

original_gldm_GrayLevelNonUniformity

original_gldm_GrayLevelVariance

original_gldm_HighGrayLevelEmphasis

original_gldm_LargeDependenceEmphasis

original_gldm_LargeDependenceHighGrayLevelEmphasis

original_gldm_LargeDependenceLowGrayLevelEmphasis

original_gldm_LowGrayLevelEmphasis

original_gldm_SmallDependenceEmphasis

original_gldm_SmallDependenceHighGrayLevelEmphasis

original_gldm_SmallDependenceLowGrayLevelEmphasis

original_glrlm_GrayLevelNonUniformity

original_glrlm_GrayLevelNonUniformityNormalized

original_glrlm_GrayLevelVariance

original_glrlm_HighGrayLevelRunEmphasis

original_glrlm_LongRunEmphasis

original_glrlm_LongRunHighGrayLevelEmphasis

original_glrlm_LongRunLowGrayLevelEmphasis

original_glrlm_LowGrayLevelRunEmphasis

original_glrlm_RunEntropy

original_glrlm_RunLengthNonUniformity

original_glrlm_RunLengthNonUniformityNormalized

original_glrlm_RunPercentage

original_glrlm_RunVariance

original_glrlm_ShortRunEmphasis

original_glrlm_ShortRunHighGrayLevelEmphasis

original_glrlm_ShortRunLowGrayLevelEmphasis

original_glszm_GrayLevelNonUniformity

original_glszm_GrayLevelNonUniformityNormalized

original_glszm_GrayLevelVariance

original_glszm_HighGrayLevelZoneEmphasis

original_glszm_LargeAreaEmphasis

original_glszm_LargeAreaHighGrayLevelEmphasis

original_glszm_LargeAreaLowGrayLevelEmphasis

original_glszm_LowGrayLevelZoneEmphasis

original_glszm_SizeZoneNonUniformity

original_glszm_SizeZoneNonUniformityNormalized

original_glszm_SmallAreaEmphasis

original_glszm_SmallAreaHighGrayLevelEmphasis

original_glszm_SmallAreaLowGrayLevelEmphasis

original_glszm_ZoneEntropy

original_glszm_ZonePercentage

original_glszm_ZoneVariance

original_ngtdm_Busyness

original_ngtdm_Coarseness

original_ngtdm_Complexity

original_ngtdm_Contrast

original_ngtdm_Strength

wavelet-LH_firstorder_10Percentile

wavelet-LH_firstorder_90Percentile

wavelet-LH_firstorder_Energy

wavelet-LH_firstorder_Entropy

wavelet-LH_firstorder_InterquartileRange

wavelet-LH_firstorder_Kurtosis

wavelet-LH_firstorder_Maximum

wavelet-LH_firstorder_MeanAbsoluteDeviation

wavelet-LH_firstorder_Mean

wavelet-LH_firstorder_Median

wavelet-LH_firstorder_Minimum

wavelet-LH_firstorder_Range

wavelet-LH_firstorder_RobustMeanAbsoluteDeviation

wavelet-LH_firstorder_RootMeanSquared

wavelet-LH_firstorder_Skewness

wavelet-LH_firstorder_TotalEnergy

wavelet-LH_firstorder_Uniformity

wavelet-LH_firstorder_Variance

wavelet-LH_glcm_Autocorrelation

wavelet-LH_glcm_ClusterProminence

wavelet-LH_glcm_ClusterShade

wavelet-LH_glcm_ClusterTendency

wavelet-LH_glcm_Contrast

wavelet-LH_glcm_Correlation

wavelet-LH_glcm_DifferenceAverage

wavelet-LH_glcm_DifferenceEntropy

wavelet-LH_glcm_DifferenceVariance

wavelet-LH_glcm_Id

wavelet-LH_glcm_Idm

wavelet-LH_glcm_Idmn

wavelet-LH_glcm_Idn

wavelet-LH_glcm_Imc1

wavelet-LH_glcm_Imc2

wavelet-LH_glcm_InverseVariance

wavelet-LH_glcm_JointAverage

wavelet-LH_glcm_JointEnergy

wavelet-LH_glcm_JointEntropy

wavelet-LH_glcm_MCC

wavelet-LH_glcm_MaximumProbability

wavelet-LH_glcm_SumAverage

wavelet-LH_glcm_SumEntropy

wavelet-LH_glcm_SumSquares

wavelet-LH_gldm_DependenceEntropy

wavelet-LH_gldm_DependenceNonUniformity

wavelet-LH_gldm_DependenceNonUniformityNormalized

wavelet-LH_gldm_DependenceVariance

wavelet-LH_gldm_GrayLevelNonUniformity

wavelet-LH_gldm_GrayLevelVariance

wavelet-LH_gldm_HighGrayLevelEmphasis

wavelet-LH_gldm_LargeDependenceEmphasis

wavelet-LH_gldm_LargeDependenceHighGrayLevelEmphasis

wavelet-LH_gldm_LargeDependenceLowGrayLevelEmphasis

wavelet-LH_gldm_LowGrayLevelEmphasis

wavelet-LH_gldm_SmallDependenceEmphasis

wavelet-LH_gldm_SmallDependenceHighGrayLevelEmphasis

wavelet-LH_gldm_SmallDependenceLowGrayLevelEmphasis

wavelet-LH_glrlm_GrayLevelNonUniformity

wavelet-LH_glrlm_GrayLevelNonUniformityNormalized

wavelet-LH_glrlm_GrayLevelVariance

wavelet-LH_glrlm_HighGrayLevelRunEmphasis

wavelet-LH_glrlm_LongRunEmphasis

wavelet-LH_glrlm_LongRunHighGrayLevelEmphasis

wavelet-LH_glrlm_LongRunLowGrayLevelEmphasis

wavelet-LH_glrlm_LowGrayLevelRunEmphasis

wavelet-LH_glrlm_RunEntropy

wavelet-LH_glrlm_RunLengthNonUniformity

wavelet-LH_glrlm_RunLengthNonUniformityNormalized

wavelet-LH_glrlm_RunPercentage

wavelet-LH_glrlm_RunVariance

wavelet-LH_glrlm_ShortRunEmphasis

wavelet-LH_glrlm_ShortRunHighGrayLevelEmphasis

wavelet-LH_glrlm_ShortRunLowGrayLevelEmphasis

wavelet-LH_glszm_GrayLevelNonUniformity

wavelet-LH_glszm_GrayLevelNonUniformityNormalized

wavelet-LH_glszm_GrayLevelVariance

wavelet-LH_glszm_HighGrayLevelZoneEmphasis

wavelet-LH_glszm_LargeAreaEmphasis

wavelet-LH_glszm_LargeAreaHighGrayLevelEmphasis

wavelet-LH_glszm_LargeAreaLowGrayLevelEmphasis

wavelet-LH_glszm_LowGrayLevelZoneEmphasis

wavelet-LH_glszm_SizeZoneNonUniformity

wavelet-LH_glszm_SizeZoneNonUniformityNormalized

wavelet-LH_glszm_SmallAreaEmphasis

wavelet-LH_glszm_SmallAreaHighGrayLevelEmphasis

wavelet-LH_glszm_SmallAreaLowGrayLevelEmphasis

wavelet-LH_glszm_ZoneEntropy

wavelet-LH_glszm_ZonePercentage

wavelet-LH_glszm_ZoneVariance

wavelet-LH_ngtdm_Busyness

wavelet-LH_ngtdm_Coarseness

wavelet-LH_ngtdm_Complexity

wavelet-LH_ngtdm_Contrast

wavelet-LH_ngtdm_Strength

wavelet-HL_firstorder_10Percentile

wavelet-HL_firstorder_90Percentile

wavelet-HL_firstorder_Energy

wavelet-HL_firstorder_Entropy

wavelet-HL_firstorder_InterquartileRange

wavelet-HL_firstorder_Kurtosis

wavelet-HL_firstorder_Maximum

wavelet-HL_firstorder_MeanAbsoluteDeviation

wavelet-HL_firstorder_Mean

wavelet-HL_firstorder_Median

wavelet-HL_firstorder_Minimum

wavelet-HL_firstorder_Range

wavelet-HL_firstorder_RobustMeanAbsoluteDeviation

wavelet-HL_firstorder_RootMeanSquared

wavelet-HL_firstorder_Skewness

wavelet-HL_firstorder_TotalEnergy

wavelet-HL_firstorder_Uniformity

wavelet-HL_firstorder_Variance

wavelet-HL_glcm_Autocorrelation

wavelet-HL_glcm_ClusterProminence

wavelet-HL_glcm_ClusterShade

wavelet-HL_glcm_ClusterTendency

wavelet-HL_glcm_Contrast

wavelet-HL_glcm_Correlation

wavelet-HL_glcm_DifferenceAverage

wavelet-HL_glcm_DifferenceEntropy

wavelet-HL_glcm_DifferenceVariance

wavelet-HL_glcm_Id

wavelet-HL_glcm_Idm

wavelet-HL_glcm_Idmn

wavelet-HL_glcm_Idn

wavelet-HL_glcm_Imc1

wavelet-HL_glcm_Imc2

wavelet-HL_glcm_InverseVariance

wavelet-HL_glcm_JointAverage

wavelet-HL_glcm_JointEnergy

wavelet-HL_glcm_JointEntropy

wavelet-HL_glcm_MCC

wavelet-HL_glcm_MaximumProbability

wavelet-HL_glcm_SumAverage

wavelet-HL_glcm_SumEntropy

wavelet-HL_glcm_SumSquares

wavelet-HL_gldm_DependenceEntropy

wavelet-HL_gldm_DependenceNonUniformity

wavelet-HL_gldm_DependenceNonUniformityNormalized

wavelet-HL_gldm_DependenceVariance

wavelet-HL_gldm_GrayLevelNonUniformity

wavelet-HL_gldm_GrayLevelVariance

wavelet-HL_gldm_HighGrayLevelEmphasis

wavelet-HL_gldm_LargeDependenceEmphasis

wavelet-HL_gldm_LargeDependenceHighGrayLevelEmphasis

wavelet-HL_gldm_LargeDependenceLowGrayLevelEmphasis

wavelet-HL_gldm_LowGrayLevelEmphasis

wavelet-HL_gldm_SmallDependenceEmphasis

wavelet-HL_gldm_SmallDependenceHighGrayLevelEmphasis

wavelet-HL_gldm_SmallDependenceLowGrayLevelEmphasis

wavelet-HL_glrlm_GrayLevelNonUniformity

wavelet-HL_glrlm_GrayLevelNonUniformityNormalized

wavelet-HL_glrlm_GrayLevelVariance

wavelet-HL_glrlm_HighGrayLevelRunEmphasis

wavelet-HL_glrlm_LongRunEmphasis

wavelet-HL_glrlm_LongRunHighGrayLevelEmphasis

wavelet-HL_glrlm_LongRunLowGrayLevelEmphasis

wavelet-HL_glrlm_LowGrayLevelRunEmphasis

wavelet-HL_glrlm_RunEntropy

wavelet-HL_glrlm_RunLengthNonUniformity

wavelet-HL_glrlm_RunLengthNonUniformityNormalized

wavelet-HL_glrlm_RunPercentage

wavelet-HL_glrlm_RunVariance

wavelet-HL_glrlm_ShortRunEmphasis

wavelet-HL_glrlm_ShortRunHighGrayLevelEmphasis

wavelet-HL_glrlm_ShortRunLowGrayLevelEmphasis

wavelet-HL_glszm_GrayLevelNonUniformity

wavelet-HL_glszm_GrayLevelNonUniformityNormalized

wavelet-HL_glszm_GrayLevelVariance

wavelet-HL_glszm_HighGrayLevelZoneEmphasis

wavelet-HL_glszm_LargeAreaEmphasis

wavelet-HL_glszm_LargeAreaHighGrayLevelEmphasis

wavelet-HL_glszm_LargeAreaLowGrayLevelEmphasis

wavelet-HL_glszm_LowGrayLevelZoneEmphasis

wavelet-HL_glszm_SizeZoneNonUniformity

wavelet-HL_glszm_SizeZoneNonUniformityNormalized

wavelet-HL_glszm_SmallAreaEmphasis

wavelet-HL_glszm_SmallAreaHighGrayLevelEmphasis

wavelet-HL_glszm_SmallAreaLowGrayLevelEmphasis

wavelet-HL_glszm_ZoneEntropy

wavelet-HL_glszm_ZonePercentage

wavelet-HL_glszm_ZoneVariance

wavelet-HL_ngtdm_Busyness

wavelet-HL_ngtdm_Coarseness

wavelet-HL_ngtdm_Complexity

wavelet-HL_ngtdm_Contrast

wavelet-HL_ngtdm_Strength

wavelet-HH_firstorder_10Percentile

wavelet-HH_firstorder_90Percentile

wavelet-HH_firstorder_Energy

wavelet-HH_firstorder_Entropy

wavelet-HH_firstorder_InterquartileRange

wavelet-HH_firstorder_Kurtosis

wavelet-HH_firstorder_Maximum

wavelet-HH_firstorder_MeanAbsoluteDeviation

wavelet-HH_firstorder_Mean

wavelet-HH_firstorder_Median

wavelet-HH_firstorder_Minimum

wavelet-HH_firstorder_Range

wavelet-HH_firstorder_RobustMeanAbsoluteDeviation

wavelet-HH_firstorder_RootMeanSquared

wavelet-HH_firstorder_Skewness

wavelet-HH_firstorder_TotalEnergy

wavelet-HH_firstorder_Uniformity

wavelet-HH_firstorder_Variance

wavelet-HH_glcm_Autocorrelation

wavelet-HH_glcm_ClusterProminence

wavelet-HH_glcm_ClusterShade

wavelet-HH_glcm_ClusterTendency

wavelet-HH_glcm_Contrast

wavelet-HH_glcm_Correlation

wavelet-HH_glcm_DifferenceAverage

wavelet-HH_glcm_DifferenceEntropy

wavelet-HH_glcm_DifferenceVariance

wavelet-HH_glcm_Id

wavelet-HH_glcm_Idm

wavelet-HH_glcm_Idmn

wavelet-HH_glcm_Idn

wavelet-HH_glcm_Imc1

wavelet-HH_glcm_Imc2

wavelet-HH_glcm_InverseVariance

wavelet-HH_glcm_JointAverage

wavelet-HH_glcm_JointEnergy

wavelet-HH_glcm_JointEntropy

wavelet-HH_glcm_MCC

wavelet-HH_glcm_MaximumProbability

wavelet-HH_glcm_SumAverage

wavelet-HH_glcm_SumEntropy

wavelet-HH_glcm_SumSquares

wavelet-HH_gldm_DependenceEntropy

wavelet-HH_gldm_DependenceNonUniformity

wavelet-HH_gldm_DependenceNonUniformityNormalized

wavelet-HH_gldm_DependenceVariance

wavelet-HH_gldm_GrayLevelNonUniformity

wavelet-HH_gldm_GrayLevelVariance

wavelet-HH_gldm_HighGrayLevelEmphasis

wavelet-HH_gldm_LargeDependenceEmphasis

wavelet-HH_gldm_LargeDependenceHighGrayLevelEmphasis

wavelet-HH_gldm_LargeDependenceLowGrayLevelEmphasis

wavelet-HH_gldm_LowGrayLevelEmphasis

wavelet-HH_gldm_SmallDependenceEmphasis

wavelet-HH_gldm_SmallDependenceHighGrayLevelEmphasis

wavelet-HH_gldm_SmallDependenceLowGrayLevelEmphasis

wavelet-HH_glrlm_GrayLevelNonUniformity

wavelet-HH_glrlm_GrayLevelNonUniformityNormalized

wavelet-HH_glrlm_GrayLevelVariance

wavelet-HH_glrlm_HighGrayLevelRunEmphasis

wavelet-HH_glrlm_LongRunEmphasis

wavelet-HH_glrlm_LongRunHighGrayLevelEmphasis

wavelet-HH_glrlm_LongRunLowGrayLevelEmphasis

wavelet-HH_glrlm_LowGrayLevelRunEmphasis

wavelet-HH_glrlm_RunEntropy

wavelet-HH_glrlm_RunLengthNonUniformity

wavelet-HH_glrlm_RunLengthNonUniformityNormalized

wavelet-HH_glrlm_RunPercentage

wavelet-HH_glrlm_RunVariance

wavelet-HH_glrlm_ShortRunEmphasis

wavelet-HH_glrlm_ShortRunHighGrayLevelEmphasis

wavelet-HH_glrlm_ShortRunLowGrayLevelEmphasis

wavelet-HH_glszm_GrayLevelNonUniformity

wavelet-HH_glszm_GrayLevelNonUniformityNormalized

wavelet-HH_glszm_GrayLevelVariance

wavelet-HH_glszm_HighGrayLevelZoneEmphasis

wavelet-HH_glszm_LargeAreaEmphasis

wavelet-HH_glszm_LargeAreaHighGrayLevelEmphasis

wavelet-HH_glszm_LargeAreaLowGrayLevelEmphasis

wavelet-HH_glszm_LowGrayLevelZoneEmphasis

wavelet-HH_glszm_SizeZoneNonUniformity

wavelet-HH_glszm_SizeZoneNonUniformityNormalized

wavelet-HH_glszm_SmallAreaEmphasis

wavelet-HH_glszm_SmallAreaHighGrayLevelEmphasis

wavelet-HH_glszm_SmallAreaLowGrayLevelEmphasis

wavelet-HH_glszm_ZoneEntropy

wavelet-HH_glszm_ZonePercentage

wavelet-HH_glszm_ZoneVariance

wavelet-HH_ngtdm_Busyness

wavelet-HH_ngtdm_Coarseness

wavelet-HH_ngtdm_Complexity

wavelet-HH_ngtdm_Contrast

wavelet-HH_ngtdm_Strength

wavelet-LL_firstorder_10Percentile

wavelet-LL_firstorder_90Percentile

wavelet-LL_firstorder_Energy

wavelet-LL_firstorder_Entropy

wavelet-LL_firstorder_InterquartileRange

wavelet-LL_firstorder_Kurtosis

wavelet-LL_firstorder_Maximum

wavelet-LL_firstorder_MeanAbsoluteDeviation

wavelet-LL_firstorder_Mean

wavelet-LL_firstorder_Median

wavelet-LL_firstorder_Minimum

wavelet-LL_firstorder_Range

wavelet-LL_firstorder_RobustMeanAbsoluteDeviation

wavelet-LL_firstorder_RootMeanSquared

wavelet-LL_firstorder_Skewness

wavelet-LL_firstorder_TotalEnergy

wavelet-LL_firstorder_Uniformity

wavelet-LL_firstorder_Variance

wavelet-LL_glcm_Autocorrelation

wavelet-LL_glcm_ClusterProminence

wavelet-LL_glcm_ClusterShade

wavelet-LL_glcm_ClusterTendency

wavelet-LL_glcm_Contrast

wavelet-LL_glcm_Correlation

wavelet-LL_glcm_DifferenceAverage

wavelet-LL_glcm_DifferenceEntropy

wavelet-LL_glcm_DifferenceVariance

wavelet-LL_glcm_Id

wavelet-LL_glcm_Idm

wavelet-LL_glcm_Idmn

wavelet-LL_glcm_Idn

wavelet-LL_glcm_Imc1

wavelet-LL_glcm_Imc2

wavelet-LL_glcm_InverseVariance

wavelet-LL_glcm_JointAverage

wavelet-LL_glcm_JointEnergy

wavelet-LL_glcm_JointEntropy

wavelet-LL_glcm_MCC

wavelet-LL_glcm_MaximumProbability

wavelet-LL_glcm_SumAverage

wavelet-LL_glcm_SumEntropy

wavelet-LL_glcm_SumSquares

wavelet-LL_gldm_DependenceEntropy

wavelet-LL_gldm_DependenceNonUniformity

wavelet-LL_gldm_DependenceNonUniformityNormalized

wavelet-LL_gldm_DependenceVariance

wavelet-LL_gldm_GrayLevelNonUniformity

wavelet-LL_gldm_GrayLevelVariance

wavelet-LL_gldm_HighGrayLevelEmphasis

wavelet-LL_gldm_LargeDependenceEmphasis

wavelet-LL_gldm_LargeDependenceHighGrayLevelEmphasis

wavelet-LL_gldm_LargeDependenceLowGrayLevelEmphasis

wavelet-LL_gldm_LowGrayLevelEmphasis

wavelet-LL_gldm_SmallDependenceEmphasis

wavelet-LL_gldm_SmallDependenceHighGrayLevelEmphasis

wavelet-LL_gldm_SmallDependenceLowGrayLevelEmphasis

wavelet-LL_glrlm_GrayLevelNonUniformity

wavelet-LL_glrlm_GrayLevelNonUniformityNormalized

wavelet-LL_glrlm_GrayLevelVariance

wavelet-LL_glrlm_HighGrayLevelRunEmphasis

wavelet-LL_glrlm_LongRunEmphasis

wavelet-LL_glrlm_LongRunHighGrayLevelEmphasis

wavelet-LL_glrlm_LongRunLowGrayLevelEmphasis

wavelet-LL_glrlm_LowGrayLevelRunEmphasis

wavelet-LL_glrlm_RunEntropy

wavelet-LL_glrlm_RunLengthNonUniformity

wavelet-LL_glrlm_RunLengthNonUniformityNormalized

wavelet-LL_glrlm_RunPercentage

wavelet-LL_glrlm_RunVariance

wavelet-LL_glrlm_ShortRunEmphasis

wavelet-LL_glrlm_ShortRunHighGrayLevelEmphasis

wavelet-LL_glrlm_ShortRunLowGrayLevelEmphasis

wavelet-LL_glszm_GrayLevelNonUniformity

wavelet-LL_glszm_GrayLevelNonUniformityNormalized

wavelet-LL_glszm_GrayLevelVariance

wavelet-LL_glszm_HighGrayLevelZoneEmphasis

wavelet-LL_glszm_LargeAreaEmphasis

wavelet-LL_glszm_LargeAreaHighGrayLevelEmphasis

wavelet-LL_glszm_LargeAreaLowGrayLevelEmphasis

wavelet-LL_glszm_LowGrayLevelZoneEmphasis

wavelet-LL_glszm_SizeZoneNonUniformity

wavelet-LL_glszm_SizeZoneNonUniformityNormalized

wavelet-LL_glszm_SmallAreaEmphasis

wavelet-LL_glszm_SmallAreaHighGrayLevelEmphasis

wavelet-LL_glszm_SmallAreaLowGrayLevelEmphasis

wavelet-LL_glszm_ZoneEntropy

wavelet-LL_glszm_ZonePercentage

wavelet-LL_glszm_ZoneVariance

wavelet-LL_ngtdm_Busyness

wavelet-LL_ngtdm_Coarseness

wavelet-LL_ngtdm_Complexity

wavelet-LL_ngtdm_Contrast

wavelet-LL_ngtdm_Strength
